# Inhibition of Acetylcholinesterase by Novel Lupinine Derivatives

**DOI:** 10.3390/molecules28083357

**Published:** 2023-04-11

**Authors:** Igor A. Schepetkin, Zhangeldy S. Nurmaganbetov, Serik D. Fazylov, Oralgazy A. Nurkenov, Andrei I. Khlebnikov, Tulegen M. Seilkhanov, Anarkul S. Kishkentaeva, Elvira E. Shults, Mark T. Quinn

**Affiliations:** 1 Department of Microbiology and Cell Biology, Montana State University, Bozeman, MT 59717, USA; igor@montana.edu; 2 Institute of Organic Synthesis and Coal Chemistry, Karaganda 100008, Kazakhstan; nzhangeldy@yandex.ru (Z.S.N.); iosu8990@mail.ru (S.D.F.); nurkenov_oral@mail.ru (O.A.N.); anar_kish@mail.ru (A.S.K.); 3 School of Pharmacy, Medical University of Karaganda, Karaganda 100012, Kazakhstan; 4 Kizhner Research Center, Tomsk Polytechnic University, 634050 Tomsk, Russia; aikhl@chem.org.ru; 5 Laboratory of Engineering Profile NMR Spectroscopy, Sh. Ualikhanov Kokshetau University, Kokshetau 020000, Kazakhstan; tseilkhanov@mail.ru; 6 N.N. Vorozhtsov Novosibirsk Institute of Organic Chemistry, Siberian Branch of the Russian Academy of Sciences, 630090 Novosibirsk, Russia

**Keywords:** acetylcholinesterase, acetylcholinesterase inhibitor, Alzheimer’s disease, ester, lupinine, molecular docking, triazole derivative

## Abstract

Alzheimer’s disease (AD) is a neurodegenerative disease characterized by progressive memory loss and cognitive impairment due in part to a severe loss of cholinergic neurons in specific brain areas. AD is the most common type of dementia in the aging population. Although several acetylcholinesterase (AChE) inhibitors are currently available, their performance sometimes yields unexpected results. Thus, research is ongoing to find potentially therapeutic AChE inhibitory agents, both from natural and synthetic sources. Here, we synthesized 13 new lupinine triazole derivatives and evaluated them, along with 50 commercial lupinine-based esters of different carboxylic acids, for AChE inhibitory activity. The triazole derivative **15** [1*S*,9a*R*)-1-((4-(4-(benzyloxy)-3-methoxyphenyl)-1*H*-1,2,3-triazol-1-yl)methyl)octahydro-2*H*-quinolizine)] exhibited the most potent AChE inhibitory activity among all 63 lupinine derivatives, and kinetic analysis demonstrated that compound **15** was a mixed-type AChE inhibitor. Molecular docking studies were performed to visualize interaction between this triazole derivative and AChE. In addition, a structure-activity relationship (SAR) model developed using linear discriminant analysis (LDA) of 11 SwissADME descriptors from the 50 lupinine esters revealed 5 key physicochemical features that allowed us to distinguish active versus non-active compounds. Thus, this SAR model could be applied for design of more potent lupinine ester-based AChE inhibitors.

## 1. Introduction

Alzheimer’s disease (AD) and other neurodegenerative disorders are predicted to become the second leading cause of death worldwide because of the increasing elderly population in most countries. AD produces gradual cognitive dysfunction, including difficulty in making decisions, language problems, mood swings, learning, orientation, and other behavioral issues [1]. The loss of cognitive function due to AD is associated with the rapid hydrolysis of acetylcholine by cholinesterases, including acetylcholinesterase (AChE). Consequently, inhibition of AChE has been proposed to be neuroprotective [2]. Indeed, AChE inhibitors represent the first line of symptomatic drug treatment for mild-to-moderate AD. AChE inhibitors were initially utilized in the treatment of myasthenia gravis, a neuromuscular condition associated with loss of ACh receptors at the neuromuscular junction, followed by skeletal muscle weakening [3,4]. AChE also represents a therapeutic target for controlling glaucoma, Parkinson’s disease, senile dementia, myasthenia gravis, and ataxia [3].

Natural products are often used as starting points for drug discovery and have been considered as the most important resource for the identification of lead compounds due to their diverse molecular architectures and a wide range of bioactivities [5,6]. Thus, natural products represent a valuable source from which novel AChE inhibitors may be discovered [4,7], and plant alkaloids, flavonoids, chalcones, xanthones and their derivatives have been screened for AChE inhibitory activity (e.g., see [8,9,10,11,12,13]). Considering the paucity of new AChE inhibitors, we explored the possibility of developing inhibitors based on transformation of the plant alkaloid lupinine. Lupinine ([(1*R*,9a*R*)-octahydro-2*H*-quinolizin-1-yl]methane) is found mainly in Lupinus and Anabasis plants [14,15,16] and is of interest as a pharmacophore. For example, lupinine has been reported to inhibit the fungal metalloprotease Mpr1 [17]. Likewise, compounds with octahydro quinolizine nuclei have been reported as ligands of serotonin receptors 5-HT3 and 5-HT4 [18]. Similar compounds have also been shown to exhibit antimalarial [19,20], antitubercular [21], and anticholinesterase activities [22,23,24,25]. The quinolizidine nucleus of lupinine is simultaneously bulky and highly lipophilic and can be used for replacement with heterocyclic groups, or the ring could be connected with bi- and tricyclic groups to develop AChE inhibitors [25,26]. On the other hand, it should be noted that simple esters of lupinine have also been reported to exhibit some anti-AChE activity [23].

We synthesized 13 lupinine-based triazole derivatives and evaluated them, together with 50 additional commercial lupinine-based esters of different carboxylic acids, for AChE inhibitory activity. This screening resulted in the identification of some novel AChE inhibitors, with the most potent being compound **15**. Molecular docking allowed us to characterize **15** for its potential interaction with the AChE binding site. We also developed a structure-activity relationship (SAR) model to predict AChE inhibitory activity of lupinine esters.

## 2. Results and Discussion

### 2.1. Chemistry

**Scheme 1:**
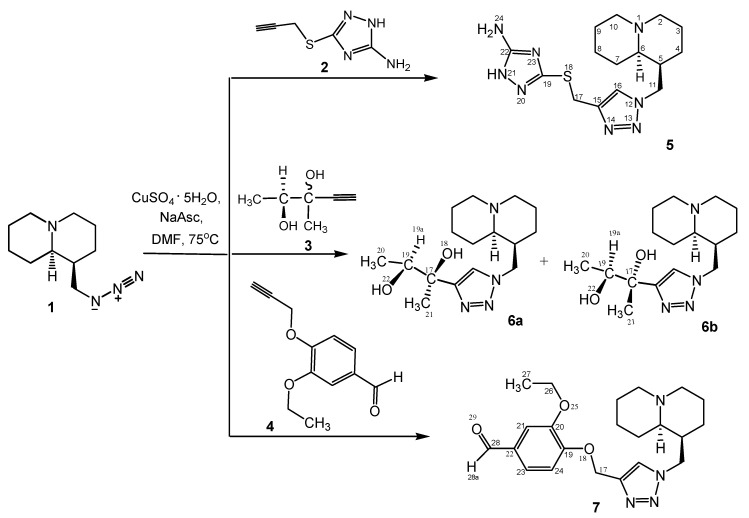
Synthesis of quinolizine-triazoles **5**–**7**.

We synthesized three novel compounds by reacting lupinine azide **1** with terminal alkynes 3-(prop-2-yn-1-yl-thio)-1*H*-1,2,4-triazole-5-amine (**2**), (2*R*,2*S*)-3-methylpent-4-yne-2,3-diol (**3**), and 3-ethoxy-4-(prop-2-ynyloxy)benzaldehyde (**4**) under the conditions of Cu-catalyzed 1,3-dipolar cycloaddition. As a result, (1*S*,9a*R*)-1-[(1,2,3-triazole-1-yl)methyl]octahydro-1*H*-quinolizines **5**–**7**, which contained various substituents at position C-4 of the 1,2,3-triazole ring were obtained (Scheme 1). Structures of the synthesized compounds **5**–**7** were confirmed by ^1^H and ^13^C NMR spectroscopy, mass spectrometry, and two-dimensional COSY (^1^H-^1^H), HMQC (^1^H-^13^C) and HMBC (^1^H-^13^C) NMR spectroscopy (see Supplementary Figures S1–S6), which established the homo- and heteronuclear spin-spin couplings. In describing the spectra, we used the numbering of core atoms shown in Scheme 1 structure **5**.

Reaction of lupinine azide **1** with a diastereomeric mixture of alkyne **3** resulted in a mixture of unseparated diastereomers **6a**,**b** (ratio 4:1, as in initial alkyne **3**). Relative stereochemistry and the ratio of diastereomers **6a**,**b** was determined by analysis of ^1^H NMR data. The proton on the substituent at C-15 of the triazole ring (H-19) appeared at 3.88 and 4.05 ppm (multiplet signals). The other triazole derivatives **8**–**17** were synthesized as single compounds, as previously described [27,28,29]; however, this is the first report of their effects on AChE activity.

### 2.2. Biological Results

We screened our library of lupinine derivatives for their effects on AChE activity in comparison with galantamine, a known AChE inhibitor used in the treatment of mild Alzheimer’s disease. The library was assembled from two sets of compounds: the first set contained 13 lupinine-based triazole derivatives **5**–**17** (Table 1), and the second set contained 50 lupinine-based esters **18**–**67** of different carboxylic acids containing aliphatic, aromatic, or heterocyclic moieties. Structures of the lupinine-based esters are shown in Supplementary Table S1. AChE inhibitory activity of all triazole derivatives, and structures of the active lupinine derivatives are shown in Table 1 and Table 2, respectively.

Due to the small number of compounds, it is difficult to draw definite conclusions on structure-activity relationships. However, it can be noted that replacing the methyl group in compound **12** with a methoxy substituent resulted in AChE inhibitory activity (compound **13**), whereas increasing the number of methoxy groups in the benzene ring to three resulted in a loss of activity (compound **14**). On the other hand, the presence of a benzyloxy group in the *para* position of the benzene ring resulted in the maximum activity among all derivatives (compound **15**; IC_50_ = 7.2 µM). Compound **15** has a 4-benzyloxyphenyl moiety, which was also present on several other previously reported AChE inhibitors, including compounds **A**–**C** with IC_50_ values in the micromolar/submicromolar range [30,31,32] (see chemical structures and AChE inhibitory activity in Table 3). AChE inhibitory activity of compound **15** was comparable to that of galantamine (IC_50_ = 8.2 ± 1.3 μM).

A visual inspection of the ester lupinine derivatives showed that a compound should contain a sufficiently bulky R group (e.g., compounds **25**, **44**, and **64**), or the linker between the ester oxygen and the terminal hydrophobic moiety should consist of four chemical bonds (e.g., compounds **22**, **43**, and **49**) for it to exhibit AChE inhibitory activity. Compound **25** (IC_50_ = 24.4 µM) bearing a 6,6-dimethyl-6,7-dihydrobenzofuran-4(5*H*)-one group was the most active of the lupinine-based esters. Noticeably, the 3,3-dimethylcyclohexanone substructure in this fragment was also present in previously reported AChE inhibitors [33].

**Figure 1:**
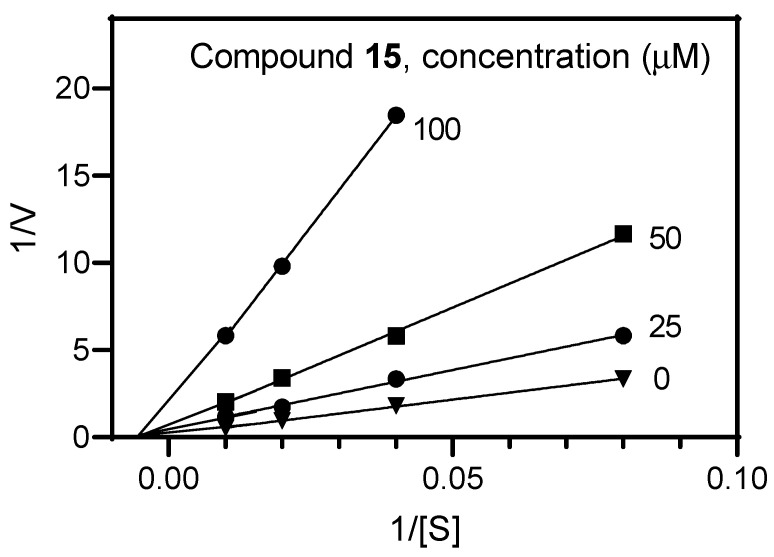
Representative double-reciprocal Lineweaver–Burk plot illustrating the mixed-type mechanism of AChE inhibition by compound **15**.

The mechanism of AChE inhibition was determined for the most active compound **15**. The Lineweaver–Burk reciprocal plot (Figure 1) revealed a series of lines converging on the same point near the x-axis, indicating that **15** caused a mixed type of inhibition, as expected for dual binding site inhibitors of AChE [34,35].

**Table 3:** Chemical structures and AChE inhibitory activities of lupinine derivative **15** and previously reported AChE inhibitors with a 4-benzyloxyphenyl moiety [36,37,38].

Name	Chemical Structure	IC_50_ (μM)
**Compound 15**	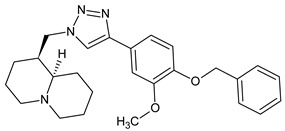	7.2
**Compound A**	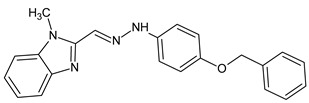	11.8
**Compound B**	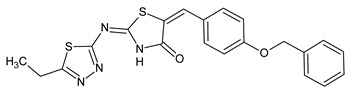	1.2
**Compound C**	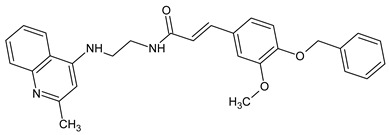	0.6

**Chemical names**: Compound **15**, (1*S*,9a*R*)-1-((4-(4-(benzyloxy)-3-methoxyphenyl)-1*H*-1,2,3-triazol-1-yl)methyl)octahydro-2*H*-quinolizine; Compound **A**, (*E*)-2-((2-(4-(benzyloxy)phenyl)hydrazineylidene)methyl)-1-methylbenzimidazole; Compound **B**, (*E*)-5-((*E*)-4-(benzyloxy)benzylidene)-2-((5-ethyl-1,3,4-thiadiazol-2-yl)imino)thiazolidin-4-one; Compound **C**, (*E*)-3-(4-(benzyloxy)-3-methoxyphenyl)-N-(2-((2-methylquinolin-4-yl)amino)ethyl)acrylamide.

Lupinine and all synthesized derivatives (**5**–**17**) were evaluated for their cytotoxicity in vitro using human THP-1 monocytic cells. These compounds had no cytotoxicity when tested at concentrations up to 50 μM. Thus, the lupinine derivatives reported here could be used for further biological evaluation in cell culture and in vivo models.

### 2.3. Molecular Docking

**Figure 2:**
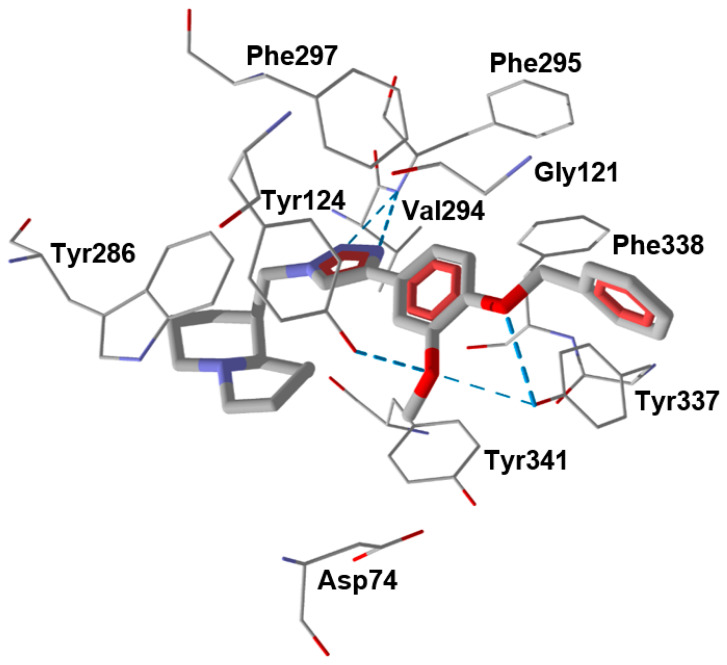
Docking poses of compound **15** in AChE (PDB code 4EY7). H-bonds are shown in blue dashed lines. Residues within 2.5 Å of each pose are visible.

We performed molecular docking of compound **15** into the AChE binding site (PDB code 4EY7) using the Rosetta ligand docking protocol implemented in the ROSIE server, which accounts for full flexibility of the main chain and side-chain residues in the vicinity of the docking area [36]. According to our modeling results, the best docking pose of compound **15** had a calculated interface energy of −24.05 kcal/mol. In this pose, the ligand forms H-bonds with residues Tyr337 (with the participation of both benzyloxy and methoxy oxygen atoms), Tyr124 (with methoxy oxygen), and Phe295 (with two nitrogen atoms of the triazole heterocycle) (Figure 2). These general features of the ligand-binding site interactions can be responsible for the AChE inhibitory activity of compound **15**. For reference, the 2D diagram of ligand-receptor interactions obtained on docking of compound **15** into AChE is shown in Supplementary Figure S7.

**Figure 3:**
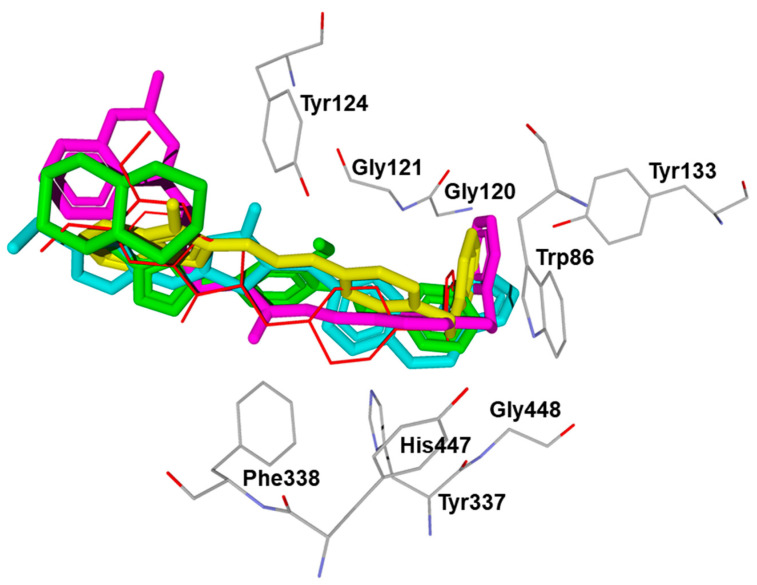
Superimposed docking poses of compounds **15** (green), **A** (yellow), **B** (light blue), and **C** (magenta) in AChE (PDB code 4EY7). The co-crystallized ligand donepezil is shown in thin red sticks. The residues embracing the hydrophobic pocket of AChE are visible. The positions of the co-crystallized donepezil and AChE residues correspond to their “native” location in the 4EY7 structure.

For comparative purposes, we modeled three other previously reported AchE inhibitors (**A**–**C**) with molecular topology analogous to compound **15** [36,37,38] (Table 3). The docking computations for these ligands led to docking poses positioned within the AChE binding site similarly to **15** (Figure 3), and with interface energies indicating strong binding: −23.34 (compound **A**), −24.70 (compound **B**), and −23.65 kcal/mol (compound **C**).

It is noteworthy that the 4-benzyloxyphenyl moieties of all docked inhibitors and the N-benzylpiperidine fragment of the co-crystallized ligand donepezil occupy the same area of space in the hydrophobic pocket surrounded by residues Trp86, Gly120, Gly121, Tyr124, Tyr133, Tyr337, Phe338, His447, and Gly448 (Figure 2 and Figure 3), although the H-bonding patterns of **A**–**C** differed from those of compound **15**. These molecules formed H-bonds with Tyr124 (compounds **A** and **C**), Tyr337 (compound **A**), His447, Ser293, and Arg296 (compound **B**). In addition, the terminal cyclic moieties of these inhibitors, including the quinolizidine heterocycle of compound **15** and the indanone fragment of donepezil, match well with each other in the AChE binding site. It should be noted that for the investigated compounds, most of the above-mentioned residues surrounding the pocket are among the top ten residues tightly interacting with the ligands, according to the partial MolDock scores as evaluated by the “Energy Inspector” tool of Molegro 6.0 software, which is due to significant van der Waals interactions of the molecules with these residues. In terms of the reported AChE functional domains [37], the subpocket residues identified belong to important functional domains, including the catalytic triad (His447), the anionic domain (Trp86, Tyr337, Phe338), and the oxyanion domain (Gly121). Additionally, we obtained high partial MolDock scores for Tyr124 and Trp286, which are located in the peripheral anionic site at the entrance of the binding gorge [37].

### 2.4. Classification SAR Model

Lupinine derivatives containing an ester moiety are, in general, more synthetically accessible. Thus, we used the lupinine ester derivatives to build an SAR model using linear discriminant analysis (LDA) to determine if it would be helpful for further drug design within this subgroup of substituted lupinines. LDA is a statistical technique used to categorize data points into two or more classes using a linear formalism [38]. The compounds containing an ester or carbamate moiety were separated into two classes (“Active” and “NA”) according to the data shown in Table 2, which includes only active AChE inhibitors found within the entire set of the lupinine esters (see Supplementary Table S1). Selected physicochemical and ADME parameters calculated using the SwissADME online tool were considered as independent variables (predictors) for LDA analysis along with two manually defined structural descriptors N_am_ and Q. Based on the 11 selected predictors, the LDA models in the form of classification functions (1) and (2) were built by STATISTICA 6.0 software with the “Best subset” option switched on.

We found that the best subset included 5 of the 11 descriptors (D_1_–D_5_, Table 2), which were sufficient for good LDA classification of the compounds, with 41 of the 50 lupinine derivatives classified correctly as AChE inhibitors (the class “Active”) or inactive compounds (the class “NA”). The values of SwissADME descriptors appearing in the classification functions are shown in Table S2 (see Supplementary Materials).

The best subset of predictors included molecular weight (MW), number of rotatable bonds (N_rot_), molar refractivity (MR), water solubility measure SILICOS-IT Log S_w_ (sLogS) [39], and the indicator Q of the quaternary carbon atom. This relatively simple LDA model can be expressed by the following two classification functions:

F(Active) = a_0_ + a_1_·D_1_ + a_2_·D_2_ + … + a_5_·D_5_ (1)

F(NA) = b_0_ + b_1_·D_1_ + b_2_·D_2_ + … + b_5_·D_5_ (2)

where D_1_–D_5_ are the values of descriptors from the best subset; a_0_, b_0_ are the intercepts from Table 4; a_1_–a_5_, b_1_–b_5_ are coefficients of the linear classification functions from the corresponding columns of Table 4.

According to the LDA model, a compound is classified as active if F (Active) > F (NA), and vice versa. Hence, the influence of each predictor can be evaluated based on the corresponding pair coefficients in the two classification functions. For example, a higher molecular weight favors activity because the coefficient for MW is larger in F (Active). The same refers to molar refractivity and water solubility. Conversely, higher molecular flexibility and the presence of a quaternary carbon atom disfavor activity in view of lower (more negative) coefficients for N_rot_ and Q predictors in F (Active).

The classification matrix for the investigated compounds is shown in Table 5. According to this matrix, the LDA model correctly classifies 6 of 7 (85.7%) active AChE inhibitors and 35 of 43 (81.4%) inactive compounds. In spite of the noticeable number of false positives among the “NA” class, a total of 82.0% of the compounds were recognized properly by the model. The per-compound classifications are presented in Table S3 (see Supplementary Materials).

The single compound which was erroneously classified as inactive (**19**) contains a chorine atom at the terminal position of the ester tail. This is a significant structural difference from other active lupinine derivatives, which contain cyclic substructures at the terminal position of each molecule.

Leave-one-out (LOO) validation of the model (i.e., predicting the activity of a discarded compound by a model built on the basis of the remaining 49 molecules) showed that 32 out of 43 inactive compounds (74.1%) and 5 out of 7 active compounds (71.4%) were correctly predicted (74 % in the total set).

The SAR model based on physicochemical descriptors of the lupinine-based esters revealed key features distinguishing AChE inhibitors versus non-active compounds. One of the weak points of the model is the imbalanced character of the data set, which contained many more inactive compounds than active ones. However, the reasonable quality and predictive ability of the model, as well as the simplicity and rapidity of the calculations associated with the LDA algorithm, suggest promise in using this model for large database mining and virtual screening of lupinine-based AChE inhibitors.

## 3. Experimental Section

### 3.1. Chemistry

^1^H and ^13^C NMR spectra were recorded on a JNN-ECA Jeol 400 spectrometer (frequency 399.78 and 100.53 MHz, respectively) with deuterated dimethyl sulfoxide (DMSO-d_6_) as the solvent. The chemical shifts were measured with reference to signals of the residual protons or carbon atoms of DMSO-d_6_. The multiplicity of signals in the ^13^C NMR spectra was determined from spectra recorded in the J-modulation mode (JMOD). The assignment of signals in the ^1^H and ^13^C NMR spectra were confirmed by two-dimensional homonuclear (^1^H-^1^H COSY) and heteronuclear ^1^H-^13^C (HMBC, HSQC) spectroscopy and literature data for quinolizine. High-resolution mass spectra were recorded on a ThermoScientific DFS spectrometer (evaporator temperature of 200–250 °C, electron ionization 70 eV). Melting points were determined on a Mettler Toledo FP900 system. The process of chemical reactions was monitored by thin-layer chromatography (TLC) on Sorbfil UV-254 plates using CH_3_Cl and CH_3_Cl–EtOH (10:1) as eluents. The plates were visualized with iodine vapor and ultraviolet (UV) light (254 nm). The reaction products were isolated by recrystallization or column chromatography using Acros silicagel (0.035–0.240 mm) and CHCl_3_ and CHCl_3_–EtOH (100:1→10:1) as eluents.

Alkynes of 3-(prop-2-yn-1-yl-thio)-1*H*-1,2,4-triazole-5-amine (**2**), (2*R*,2S)-3-methylpent-4-yne-2,3-diol (4:1, diastereomeric mixture) (**3**) and 3-ethoxy-4-(prop-2-ynyloxy)benzaldehyde (**4**) were purchased from Alfa Aesar.

Compounds **1** and **8**–**17** were synthesized as described previously [27,28,29]. The synthesized structures were confirmed by analytical and spectral data. Sample purity was >99%.

(–)-Lupinine (m.p. 69–71 °C (EtOH), [α]_D_^25^–30.5° (*c* 0.41, MeOH); (literature data: m.p. 68–69 °C (EtOH), [α]_D_^25^–23.5°) [40] was isolated from the *Anabasis aphyla* L., as described previously [41].

Lupinine azide **1** was obtained from lupinine in two stages, as described previously [28]. Briefly, the reaction of (–)-lupinine with methanesulfonyl chloride in the presence of Et_3_N in CH_2_Cl_2_ resulted in (1*R*,9a*R*)-(octahydro-2*H*-quinolizine-1-yl)methyl methanesulfonate, which was treated with NaN_3_ in dimethylformamide (DMF), resulting in the organic quinolizine azide (**1**) [29].

#### 3.1.1. General Procedure for Compounds (**5**–**7**)

A mixture of lupinine azide (**1**) (0.29 g, 1.5 mmol), substituted acetylene [3-(prop-2-yn-1-yl-thio)-1*H*-1,2,4-triazole-5-amine (**2**), (2*S*)-3-methylpent-4-yne-2,3-diol (**3**), and 3-ethoxy-4-(prop-2-ynyloxy)benzaldehyde (**4**) (1.35 mmol), CuSO_4_ × 5H_2_O (0.017 g, 0.0675 mmol) and sodium ascorbate (0.013 g, 0.0675 mmol) in DMF (6 mL) was stirred at 75 °C for 6–8 h using TLC monitoring. After cooling, the residue was filtered, washed with hexane, and dried. Triazoles **5**–**7** were isolated from the residue by chromatography on silicagel (eluent: CH_3_Cl, CH_3_Cl–EtOH, 100:1 → 10:1).

#### 3.1.2. 3-((1-(((1*S*,9a*R*)-Octahydro-1*H*-quinolizine-1-yl)methyl)-1*H*-1,2,3-triazole-4-yl)methylthio)-1*H*-1,2,4-triazole-5-amine (**5**)

Yield 0.22 g (75.86%). Dark-brown powder, m.p. 177–179 °C (decomp.). ^1^H NMR spectrum (DMSO-d_6_), δ, ppm: 1.15–1.66 m (10H, H-3ax,3eq, 4ax,4eq,7ax,7eq,8ax,8eq,9ax,9eq), 1.89 s (2H, H-2ax,10ax), 2.05 s (2H, H-5,6), 2.75 s (2H, H-2eq,10eq), 4.19 s (2H, H-17,17), 4.41 s (2H, H-11,11), 5.97 s (2H, H-24,24), 7.90 s (1H, H-16), 11.95 br. s (1H, H-21). ^13^C NMR spectrum (DMSO-d_6_), δ, ppm: 20.67 (C-3), 24.70 (C-8,9), 26.73 (C-17), 28.85 (C-4,7), 39.27 (C-5), 48.76 (C-11), 57.38 (C-2,10), 64.17 (C-6), 124.09 (C-16), 144.44 (C-15) and 156.20 (C-19,22). Mass spectrum, *m*/*z* (*I*_rel._, %) (**2**): 348.2 (7.23), 232.2 (17.40), 151.1 (100.0), 96.0 (21.73), 55.0 (16.38). Found *m*/*z*: 348.1839 [M]^+^. C_15_H_24_N_8_S. Calculated *m*/*z*: 348.1838.

#### 3.1.3. (2*R*,*S*)-2-(1-(((1*S*,9a*R*)-Octahydro-1*H*-quinolizine-1-yl)methyl)-1*H*-1,2,3-triazole-4-yl)butane-2,3-diol (**6**)

Yield 0.25 g (86.20%). Cream-colored, m.p. 158–161 °C. ^1^H NMR (DMSO-d_6_), δ, ppm: 1.03 d (3H, H-22,22,22), 1.04–1.95 m (10H, H-3ax,3eq, 4ax,4eq,7ax,7eq,8ax,8eq,9ax,9eq), 1.52 s (3H, H-21,21,21), 1.93–1.98 m (2H, H-2ax,10ax), 1.97–2.06 m (1H, H-6), 2.15–2.21 m (1H, H-5), 2.81 s (2H, H-2eq,10eq), 3.41 br. s (2H, H-18,22), 4.46–4.57 m (2H, H-11,11), 7.47 s (1H, H-16). ^13^C NMR (DMSO-d_6_), δ, ppm: 17.86 (C-22), 22.98 (C-21), 24.32 (C-3), 24.88 (C-8), 25.38 (C-9), 26.20 (C-4), 29.73 (C-7), 39.26 (C-5), 57.23 (C-2,10), 64.27 (C-6), 73.07 (C-17), 74.54 (C-19), 121.89 (C-16), 151.81 (C-15). Mass spectrum, *m*/*z* (*I*_rel._, %): 308.3 (12.45), 219.2 (1.66), 151.1 (100.0), 98.0 (9.77), 43.2 (6.68). Found *m*/*z*: 308.2207 [M]^+^. C_16_H_28_N_4_O_2_. Calculated *m*/*z*: 308.2211.

#### 3.1.4. 3-Ethoxy-4-((1-(((1*S*,9a*R*)-octahydro-1*H*-quinolizine-1-yl)methyl)-1*H*-1,2,3-triazole-4-yl)methoxy)benzaldehyde (**7**)

Yield 0.23 g (76.66%). White powder, m.p. 166–168 °C. ^1^H NMR (DMSO-d_6_), δ, ppm: 1.12–1.23 m (3H, H-4ax, H-7ax, H-3ax), 1.27 t (3H, H-27,27,27, ^3^J 7.6 Hz), 1.34–1.41 m (2H, H-4eq, H-7eq), 1.46–1.49 m (2H, H-8ax, H-8eq), 1.47–1.51 m (2H, H-11,11), 1.63–1.74 m (3H, H-2ax, H-10ax, H-3eq), 1.92–1.95 m (1H, H-6), 2.70–2.72 m (2H, H-2eq, H-10eq), 2.09 br. s (1H, H-5), 4.03 q (2H, H-26,26, ^3^J 7.6 Hz), 5.23 s (2H, H-17,17), 7.32 d (1H, H-24, ^3^J 9.2 Hz), 7.48 d (1H, H-23, ^3^J 9.2 Hz), 8.24 s (1H, H-16), 9.79 s (1H, H-28). ^13^C NMR (DMSO-d_6_), δ, ppm: 15.13 (C-27), 21.82 (C-3), 25.03 (C-8), 25.77 (C-9), 25.98 (C-4), 26.36 (C-7), 39.07 (C-5), 57.09 (C-2,10), 48.34 (C-11), 62.60 (C-17), 64.67 (C-6), 64.86 (C-26), 111.57 (C-21), 113.4 (C-24), 126.26 (C-23,16), 130.49 (C-22), 149.09 (C-19), 153.50 (C-20), 191.96 (C-28). Mass spectrum, *m*/*z* (*I*_rel._, %): 398.3 (28.87), 256.2 (4.49), 151.1 (100.0), 84.9 (24.34), 55.0 (23.62). Found *m*/*z*: 398.2312 [M]^+^. C_22_H_30_N_4_O_3_. Calculated *m*/*z*: 398.2314.

### 3.2. Commercial Compounds

Fifty lupinine-based esters of different carboxylic acids (compounds **18**–**67**) were purchased from the Vitas-M laboratory (Champaign, IL, USA). All compounds were dissolved in DMSO at a stock concentration of 10 mM and stored at −20 °C.

### 3.3. AChE Inhibition Assay

The inhibitory effect of test compounds and galantamine (Tocris Bioscience, San Francisco, CA, USA) on AChE activity was determined using an AChE inhibitor screening kit from the Sigma-Aldrich Chemical Co., (St. Louis, MO, USA). The kit is based on an improved Ellman method, whereby thiocholine produced from AChE activity forms a yellow color with 5,5′-dithiobis(2-nitrobenzoic acid), and the intensity the color (412 nm) is proportional to the enzyme activity. The concentration of compound required to cause 50% inhibition (IC_50_) was determined by graphing the % inhibition of enzyme activity versus the logarithm of concentration of the test compound using 5–7 tested concentrations.

### 3.4. Cytotoxicity Assay

The cytotoxicity of the synthesized compounds was analyzed using a CellTiter-Glo Luminescent Cell Viability Assay Kit (Promega, Madison, WI, USA) according to the manufacturer’s protocol. Human THP-1 monocytic cells obtained from ATCC (Manassas, VA, USA) were cultured in RPMI 1640 medium (Mediatech Inc., Herndon, VA, USA) supplemented with 10% (*v*/*v*) FBS, 100 μg/mL streptomycin, and 100 U/mL penicillin. For the cytotoxicity assay, the cells were cultured at a density of 10^4^ cells/well with different concentrations of the test compounds added (3, 6.125, 12.5, 25, 50 μM; final concentration of DMSO was 1%) for 24 h at 37 °C and 5% CO_2_. Following treatment, substrate was added to the cells, and the samples were analyzed with a Fluoroscan Ascent FL microplate reader.

### 3.5. Molecular Docking

Docking of compounds into the acetyl cholinesterase binding site (structure 4EY7 from the Protein Data Bank) was performed using the ROSIE server [42]. The docking area was chosen around the geometric center of co-crystallized donepezil (1-benzyl-4-[(5,6-dimethoxy-1-indanon-2-yl)methyl]piperidine) occupying the binding site of the receptor in the 4EY7 structure. For each of the investigated compounds, up to 2000 ligand conformers were generated with the BCL algorithm [43] switched on. The number of intermediately generated docking poses was set to 2000. Other options were used as defaults within the ROSIE ligand docking protocol, which accounted for full flexibility of the main chain and side-chains for residues in the vicinity of the docking area [36]. After completion of the computations, PDB files containing the best poses of compounds docked into AChE were downloaded from the server and imported into Molegro Virtual Docker 6.0 (MVD) for visualization and analysis using the built-in “Pose Organizer” tool of MVD.

### 3.6. Linear Discriminant Analysis (LDA)

Structures of the 50 lupinine-based esters were built using ChemOffice 2016, represented as SMILES strings, and imported into the SwissADME online tool [39]. The calculated physicochemical and ADME parameters were subjected to correlation analysis to select descriptors with low mutual pairwise correlations. The following descriptors were selected: molecular weight (MW), fraction of sp^3^ carbon atoms (Csp3), number of rotatable bonds (N_rot_), number of hydrogen bond donors and acceptors (NHBD and NHBA, respectively), molar refractivity (MR), topological polar surface area (tPSA), consensus LogP (cLogP), and water solubility SILICOS-IT Log S_w_ (sLogS). Two structural indicators were added, which indicated absence or presence (value of 0 or 1, respectively) of an amide unit -C(O)NH- or a quaternary sp^3^ carbon atom (descriptors N_am_ and Q, respectively). Although useful computational methods have been developed for finding molecular subunits (e.g., [44]), the N_am_ and Q indicators were assigned manually because of their simplicity. The data sheet containing columns with the values of the independent predictors enumerated above was supplemented with a column indicating compound activity (values “Active” or “NA”) as a categorical dependent variable. The resulting data sheet was imported in STATISTICA 6.0 program (StatSoft, Inc., Tulsa, OK, USA), and the LDA procedure was performed with the “Best subset” option switched on using equal prior probabilities for the compound classes. All 50 lupinine-based esters were used as a training set. To validate the model, the leave-one-out (LOO) procedure was performed by sequentially discarding one of the compounds and predicting its activity class (i.e., the dependent categorical variable) by an LDA model obtained on the basis of the remaining 49 compounds.

## 4. Conclusions

We identified compound 15 as a novel AChE inhibitor and showed that it exhibited mixed-type inhibitory activity. Molecular docking modeling indicated that compound 15 meets structural requirements necessary to reproduce important intermolecular interactions described in the literature as fundamental for AChE inhibition. Thus, this compound could be a promising candidate for evaluation in AD models. Our results also indicate that the 4-benzyloxyphenyl moiety attached to different molecular scaffolds can play an important role in ligand binding to AChE due to the interaction with the receptor subpocket. This finding, as well as the derived classification SAR model, may be useful in the design of other novel AChE inhibitors.

## Figures and Tables

**Table 1:**
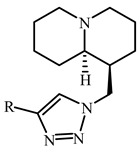
AChE inhibitory activity of lupinine-based triazole derivatives.

Compd.	R	IC_50_ (µM)	Compd.	R	IC_50_ (µM)
**5**	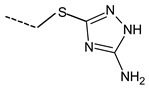	101.1 ± 23.9	**13**	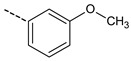	51.8 ± 9.2
**6**	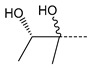	N.A.	**14**	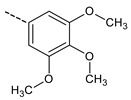	N.A.
**7**	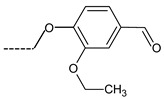	73.4 ± 16.7	**15**	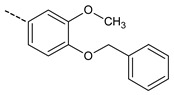	7.2 ± 0.2
**8**	---CH_2_OH	N.A.	**16**	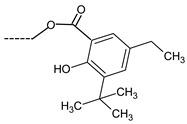	141.2 ± 15.3
**9**	---C(CH_3_)_2_OH	N.A.			
**10**	---C(CH_3_)_2_O(CH_2_)_2_CN	N.A.			
**11**	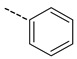	N.A.			
**12**	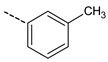	N.A.	**17**	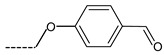	68.1 ± 2.2

Compound **6** was present as a mixture of diastereomers **6a**,**b** (ratio 4:1). N.A.: no activity was observed at the highest concentration tested (150 µM).

**Table 2:**
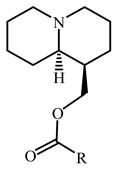
AChE inhibitory activity of lupinine-based esters of different carboxylic acids.

Compd.	R	IC_50_ (µM)	Compd.	R	IC_50_ (µM)
**22**	---(CH_2_)_3_Cl	39.5 ± 7.2	**44**	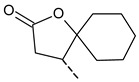	68.9 ± 5.8
**25**	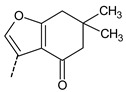	24.4 ± 3.4			
**41**	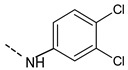	99.4 ± 12.3	**49**	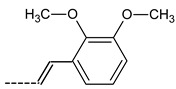	111.3 ± 12.5
**43**	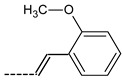	89.6 ± 7.7	**64**	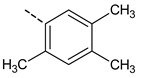	74.1 ± 6.8

**Table 4:** Physicochemical descriptors from the best subset and the corresponding coefficients of classification functions.

Descriptor		Coefficient of Classification Functions	
		Active	NA
	Intercept	−61.838	−51.489
D_1_	MW	0.064	0.046
D_2_	N_rot_	−0.848	−0.133
D_3_	MR	1.450	1.354
D_4_	sLogS	9.175	8.580
D_5_	Q	−7.863	−5.265

Abbreviations: molecular weight (MW), number of rotatable bonds (N_rot_), molar refraction (MR), water solubility characteristic (sLogS), and indicator variable for the quaternary sp^3^ carbon atom (Q).

**Table 5:** Classification matrices for the LDA model built based on 50 lupinine ester derivatives.

	Percent	Non-Active (Calculated)	Active (Calculated)
Non-active (observed)	81.4	**35**	8
Active (observed)	85.7	1	**6**
Total	82.0	36	14

The number of compounds correctly classified by the model is indicated in bold.

## Data Availability

Data are contained within the article.

## Supplementary Materials

The following supporting information can be downloaded at: https://www.mdpi.com/article/10.3390/molecules28083357/s1, Supplementary Figures S1–S3. Schemes of correlations in the COSY and HMQC spectra of **5**–**7**. Supplementary Figure S4.1. ^1^H NMR spectrum of **5**. Supplementary Figure S4.2. ^13^C NMR spectrum of **5**. Supplementary Figure S4.3. The mass spectrum of **5**. Supplementary Figure S5.1. ^1^H NMR spectrum of **6a**,**b**. Supplementary Figure S5.2. ^13^C NMR spectrum of **6a**,**b**. Supplementary Figure S5.3. The mass spectrum of **6a**,**b**. Supplementary Figure S6.1. ^1^H NMR spectrum of **7**. Supplementary Figure S6.2. ^13^C NMR spectrum of **7**. Supplementary Figure S6.3. The mass spectrum of **7**. Supplementary Table S1. Chemical structures of lupinine-based esters of different carboxylic acids under investigation. Supplementary Figure S7. 2D diagram of ligand-receptor interactions obtained on docking of compound **15** in AChE. Blue dashed lines—hydrogen bonding interactions. Red dashed line—steric interaction. Supplementary Table S2. Chemical formulas, selected ADME parameters of the lupinine-based esters calculated with SwissADME web tool, and manually added indicator variable for the quaternary sp^3^ carbon atom (Q). Supplementary Table S3. Experimentally determined, calculated, and LOO-predicted classes for AChE inhibitory activity of the lupinine-based esters of different carboxylic acids.

## Sample Availability

Samples of synthesized compounds are available from the authors.
