# Supplementary material for: Inhibition of Acetylcholinesterase by Novel Lupinine Derivatives
Source: Molecules. 2023 Apr 11;28(8):3357. doi: 10.3390/molecules28083357 (PMC10146204; doi:10.3390/molecules28083357)
Supplement: Supplementary file 1 [file molecules-28-03357-s001.zip › molecules-2316204-supplementary.pdf]

Supplementary Material

Inhibition of Acetylcholinesterase by Novel Lupinine Derivatives

Igor A. Schepetkin[1], Zhangeldy S. Nurmaganbetov[2,3], Serik D. Fazylov[2], Oralgazy A. Nurkenov[2], Andrei I. Khlebnikov[4], Tulegen M. Seilkhanov[5], Anarkul S. Kishkentaeva[2], Elvira E. Shults[6], and Mark T. Quinn[1]

[1]*Department of Microbiology and Cell Biology, Montana State University, Bozeman, MT 59717, USA*

[2]*Institute of Organic Synthesis and Coal Chemistry, Karaganda 100008, Kazakhstan*

[3]*Karaganda Medical University, Karaganda 100012, Kazakhstan*

[4]*Kizhner Research Center, Tomsk Polytechnic University, Tomsk 634050, Russia*

[5]*Sh. Ualikhanov Kokshetau University, Kokshetau 020000, Kazakhstan*

[6]*N.N. Vorozhtsov Novosibirsk Institute of Organic Chemistry, Siberian Branch of the Russian Academy of Sciences, Novosibirsk 630090, Russia*

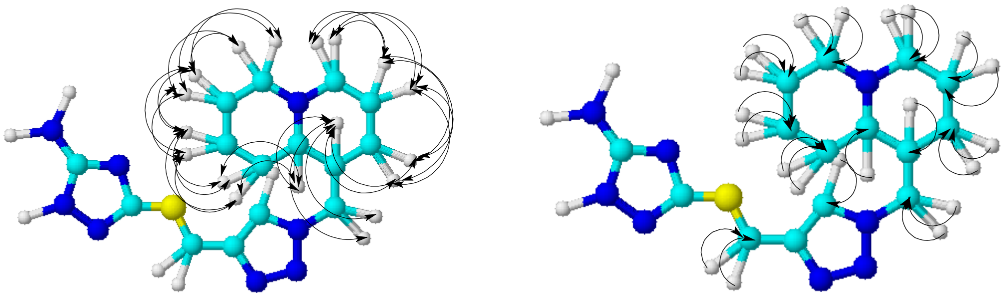

COSY (a) HMQC (b)

Supplementary Figure S1. Scheme of correlations in the COSY (a) and HMQC (b) spectra of compound 5.

^{1}H-^{1}H COSY spectra of the compound showed the spin-spin couplings through three bonds of the neighboring methine proton of the lupinine cycle with the methylene proton H^{5}-H^{11} (2.05, 4.39 and 4.39, 2.05) and the methylene-methylene protons of the lupinine cycles of $H^{2ax,10ax}$-$H^{2eq,10eq}$ (1.88, 2.73 and 2.73, 1.88) ppm. The heteronuclear couplings of protons with carbon atoms through a single bond were established by ^{1}H-^{13}C HMQC spectroscopy for the following pairs in the compound: H^{3ax}-C^{3} (1.27, 20.64), H^{4ax}-C^{4} (1.39, 29.06), $H^{2ax,10ax}$-$C^{2,10}$ (1.83, 56.82), H^{6}-C^{6} (2.02, 64.17), H^{5}-C^{5} (2.04, 39.27), $H^{2eq,10eq}$-$C^{2,10}$ (2.75, 57.36), H^{11}-C^{11} (4.40, 48.76), H^{17}-C^{17} (4.18, 26.73) and H^{16}-C^{16} (7.89, 124.17) ppm. The heteronuclear couplings of protons with carbon atoms through two or more bonds were determined by ^{1}H-^{13}C HMBC spectroscopy for the following pairs in the compound: H^{17}-C^{16} (4.17, 124.06), H^{17}-C^{15} (4.17, 144.44), H^{17}-C^{19} (4.17, 156.20); H^{11}-C^{21} (7.47, 125.65), H^{21}-C^{19} (7.47, 148.52); H^{16}-C^{16} (8.23, 125.97), H^{16}-C^{5} (4.40, 38.88); H^{16}-C^{16} (7.84, 124.32) and H^{16}-C^{15} (7.84, 144.44) ppm.

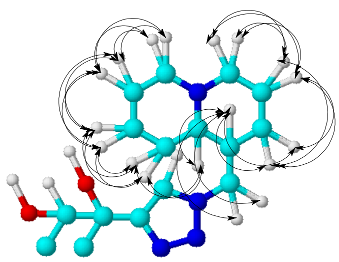

COSY (a)

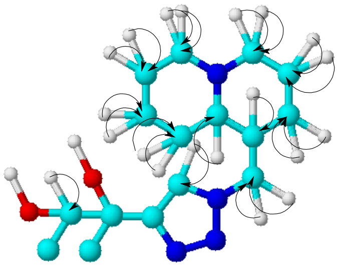

HMQC (b)

Supplementary Figure S2. Scheme of correlations in the COSY (a) and HMQC (b) spectra of compound 6.

^{1}H-^{1}H COSY spectra of the compound demonstrated the spin-spin couplings through three bonds of the neighboring methine-methylene groups of H^{5}-H^{11} (2.14, 4.50 and 4.50, 2.14) ppm. The heteronuclear couplings of protons with carbon atoms through a single bond were established by ^{1}H-^{13}C HMQC spectroscopy for the following pairs in the compound: H^{22}-C^{22} (1.02, 16.99), H^{21}-C^{21} (1.51, 23.15), H^{4ax}-C^{4} (1.19, 26.20), H^{4eq}-C^{4} (1.30, 26.30), H^{7ax}-C^{7} (1.45, 29.15), H^{7eq}-C^{7} (1.55, 29.17), $H^{2ax,10ax}$-$C^{2,10}$ (1.91, 57.53), $H^{2eq,10eq}$-$C^{2,10}$ (2.76, 57.53), H^{6}-C^{6} (2.02, 64.40), H^{5}-C^{5} (2.14, 39.43), H^{19}-C^{19} (3.88 and 4.05, 74.36 and 74.54), H^{11}-C^{11} (4.48, 48.48) and H^{16}-C^{16} (7.45, 121.95) ppm. The heteronuclear couplings of protons with carbon atoms through two or more bonds were defined by ^{1}H-^{13}C HMBC spectroscopy for the following pairs in the compound: H^{22}-C^{19} (1.06, 73.35); H^{21}-C^{17} (1.38, 73.58) and H^{21}-C^{15} (1.38, 152.77) ppm.

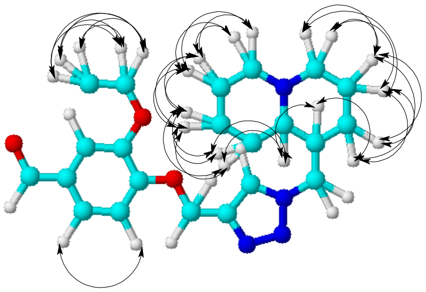

COSY (a)

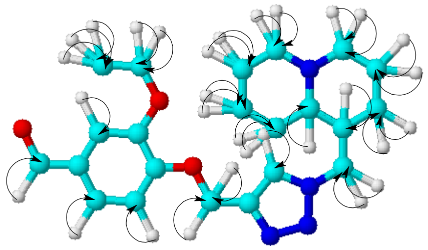

HMQC (b)

Supplementary Figure S3. Scheme of correlations in the COSY (a) and HMQC (b) spectra of compound 7.

^{1}H-^{1}H COSY spectra of the compound showed the spin-spin couplings through three bonds of the neighboring methyl and methylene groups of H^{27}-H^{26} with the cross peak at 1.25, 4.03 and 4.03, 1.25 of the ethoxy fragment of the molecule, methine proton of the lupinine cycle with methylene proton H^{5}-H^{11} (2.07, 4.48 and 4.48, 2.07), methylene-methylene protons of lupinine cycles of $H^{2ax,10ax}$-$H^{2eq,10eq}$ (1.79, 2.68 and 2.68, 1.79) and the aromatic methine protons of H^{24}-H^{23} (7.32, 7.48 and 7.48, 7.32) ppm. The heteronuclear couplings of protons with carbon atoms through a single bond were established by ^{1}H-^{13}C HMQC spectroscopy for the following pairs in the compound: H^{27}-C^{27} (1.25, 15.13), H^{3ax}-C^{3} (1.26, 20.79), H^{4ax}-C^{4} (1.28, 25.98), H^{4eq}-C^{4} (1.48, 25.98), H^{7eq}-C^{7} (1.38, 29.45), $H^{2ax,10ax}$-$C^{2,10}$ (1.78, 57.70), H^{6}-C^{6} (1.92, 64.67), H^{3eq}-C^{3} (1.70, 20.60), H^{5}-C^{5} (2.07, 39.06), $H^{2eq,10eq}$-$C^{2,10}$ (2.68, 57.33), H^{11}-C^{11} (4.48, 48.47), H^{26}-C^{26} (4.01, 64.86), H^{17}-C^{17} (5.21, 62.60), H^{21}-C^{21} (7.32, 111.57), H^{24}-C^{24} (7.31, 113.46), H^{23}-C^{23} (7.46, 126.26) and H^{16}-C^{16} (8.23, 126.26) ppm. The heteronuclear couplings of protons with carbon atoms through two or more bonds were detected by ^{1}H-^{13}C HMBC spectroscopy for the following pairs in the compound: H^{26}-C^{27} (4.00, 15.48); H^{11}-C^{5} (4.49, 39.00); H^{17}-C^{16} (5.22, 125.65), H^{17}-C^{15} (5.22, 142.72), H^{17}-C^{20} (5.22, 153.35); H^{21}-C^{21} (7.47, 125.65), H^{21}-C^{19} (7.47, 148.52); H^{16}-C^{16} (8.23, 125.97), H^{16}-C^{15} (8.23, 142.72); H^{22}-C^{21} (9.77, 111.80); H^{28}-C^{28} (9.77, 192.33) and H^{28}-C^{22} (9.77, 130.80) ppm.

Supplementary Figure S4.1. ^{1}H NMR spectrum of 3-((1-(((1*S*,9a*R*)-octahydro-1*H*-quinolizine-1-yl)methyl)-1*H*-1,2,3-triazole-4-yl)methylthio)-1*H*-1,2,4-triazole-5-amine (5).

^{1}H NMR (399.78 MHz, DMSO-d$_6$)

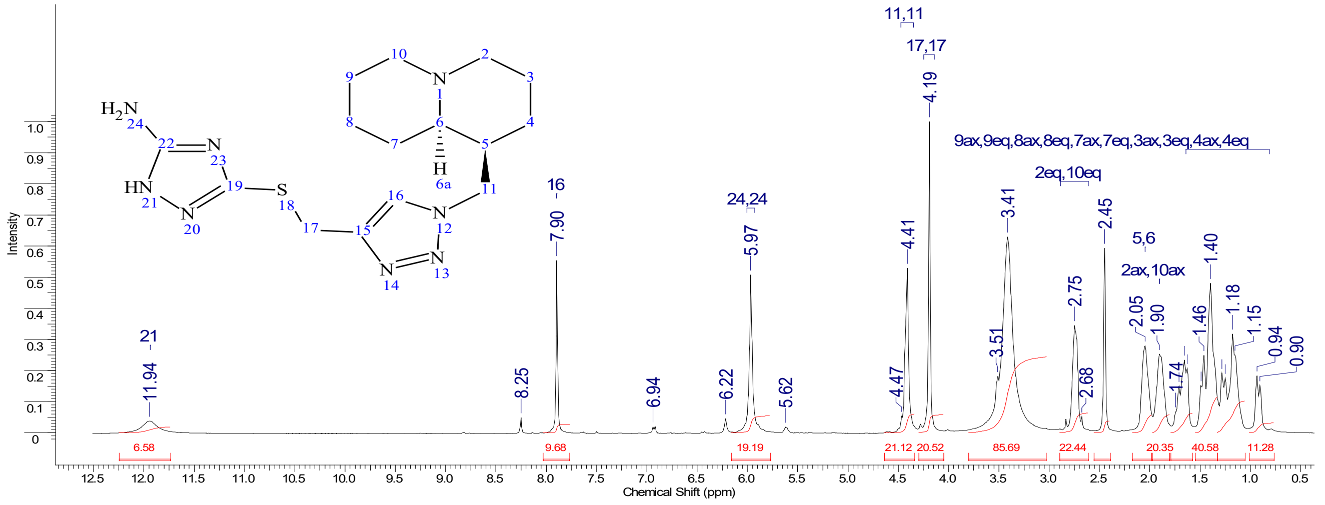

Supplementary Figure S4.2. ^{13}C NMR spectrum of 3-((1-(((1*S*,9a*R*)-octahydro-1*H*-quinolizine-1-yl)methyl)-1*H*-1,2,3-triazole-4-yl)methylthio)-1*H*-1,2,4-triazole-5-amine (5).

^{13}C NMR (100.53 MHz, DMSO-d$_6$)

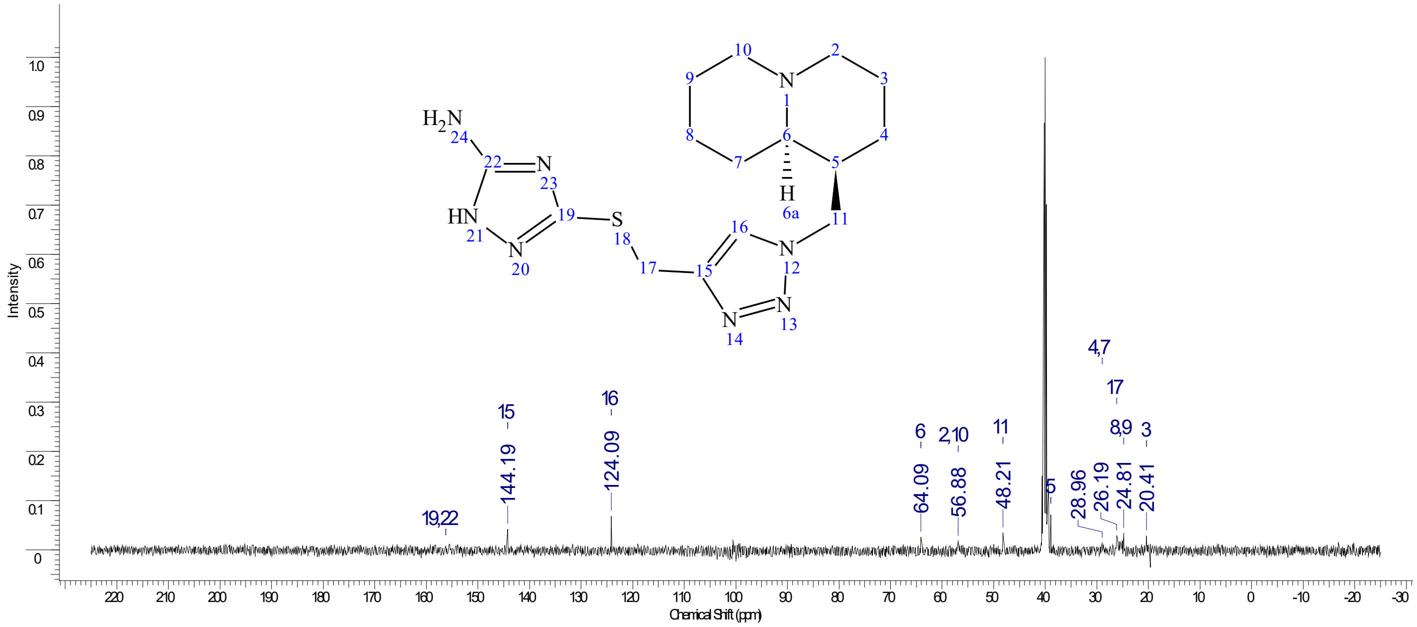

Supplementary Figure S4.3. The mass spectrum of 3-((1-(((1*S*,9a*R*)-octahydro-1*H*-quinolizine-1-yl)methyl)-1*H*-1,2,3-triazole-4-yl)methylthio)-1*H*-1,2,4-triazole-5-amine (5).

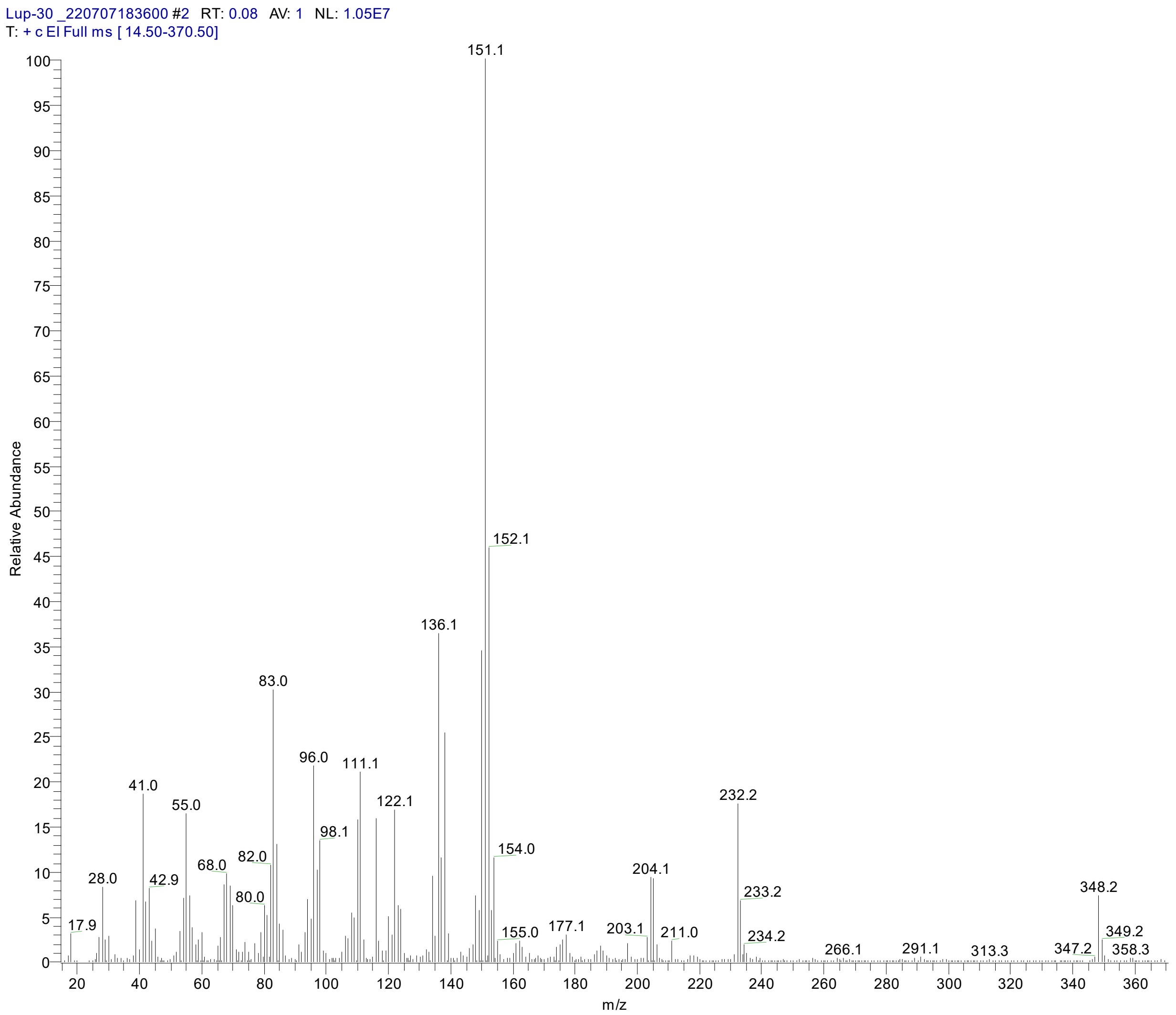

Supplementary Figure S5.1. ^{1}**H NMR spectrum of (2*R*,*S*)-2-(1-(((1*S*,9a*R*)-octahydro-1*H*-quinolizine-1-yl)methyl)-1*H*-1,2,3-triazole-4-yl)butane-2,3-diol (6a,b).**

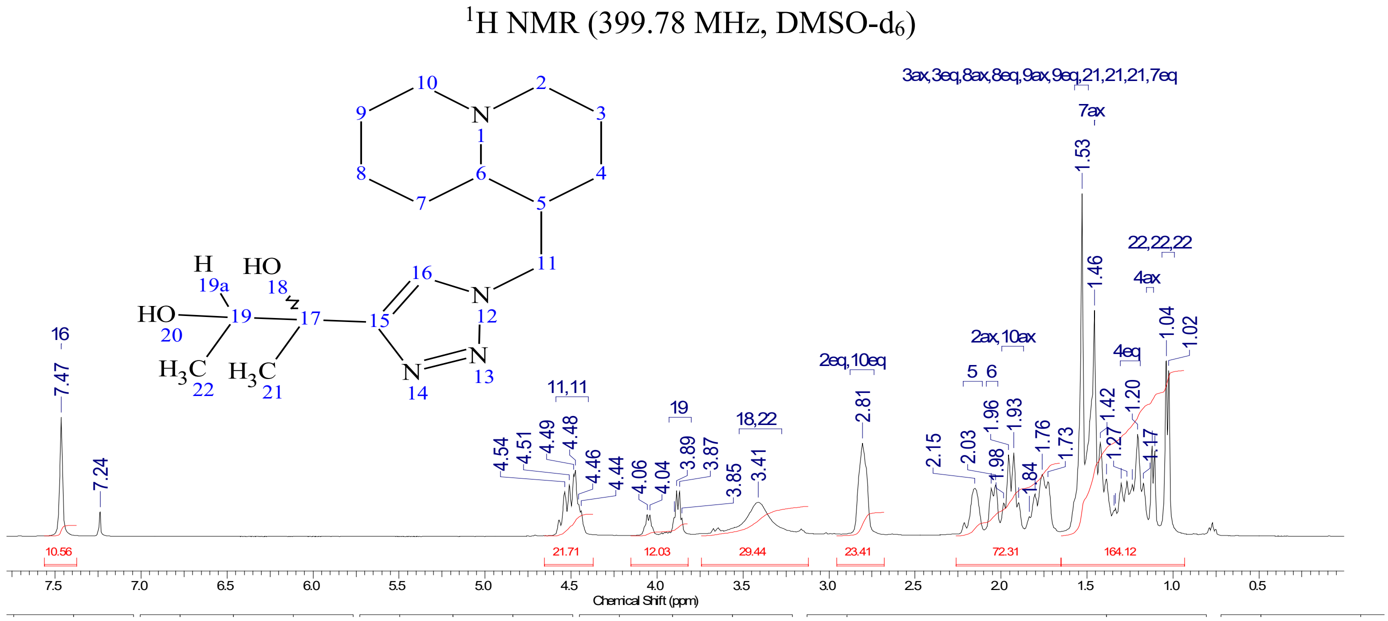

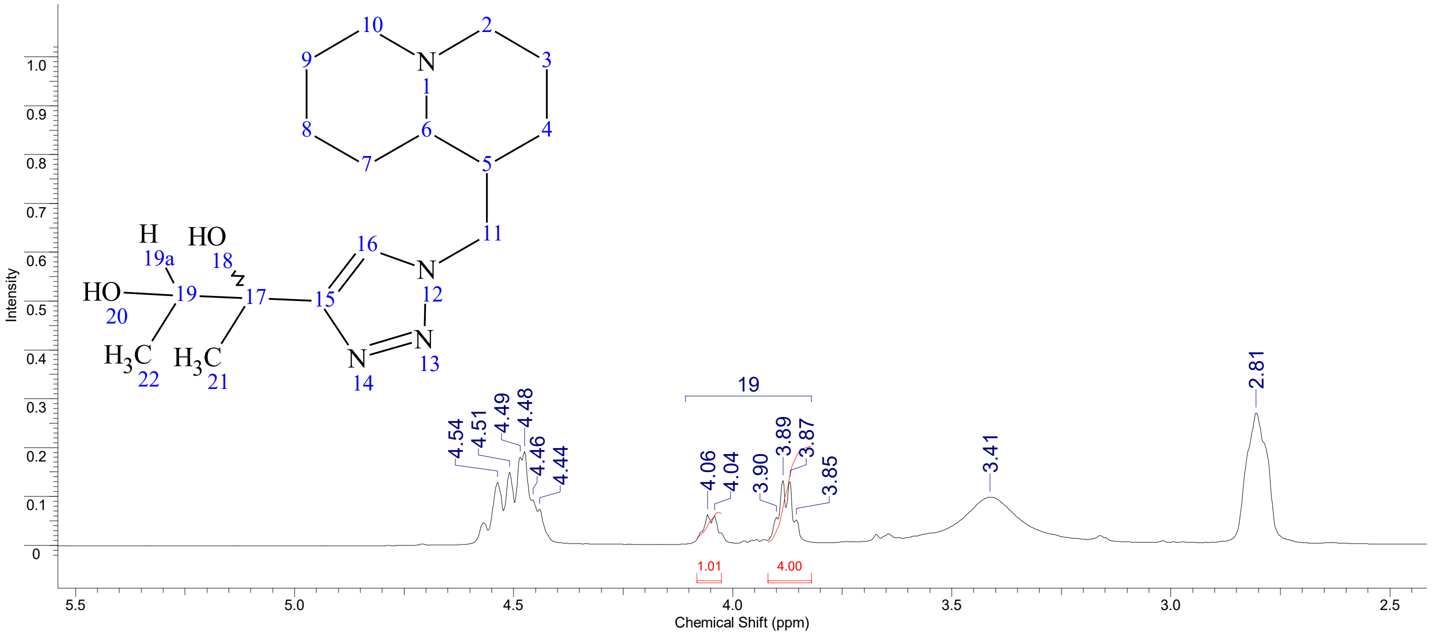

Supplementary Figure S5.2. ^{13}**C NMR spectrum of (2*R*,*S*)-2-(1-(((1*S*,9a*R*)-octahydro-1*H*-quinolizine-1-yl)methyl)-1*H*-1,2,3-triazole-4-yl)butane-2,3-diol (6a,b).**

^{13}C NMR (100.53 MHz, DMSO-d_6)

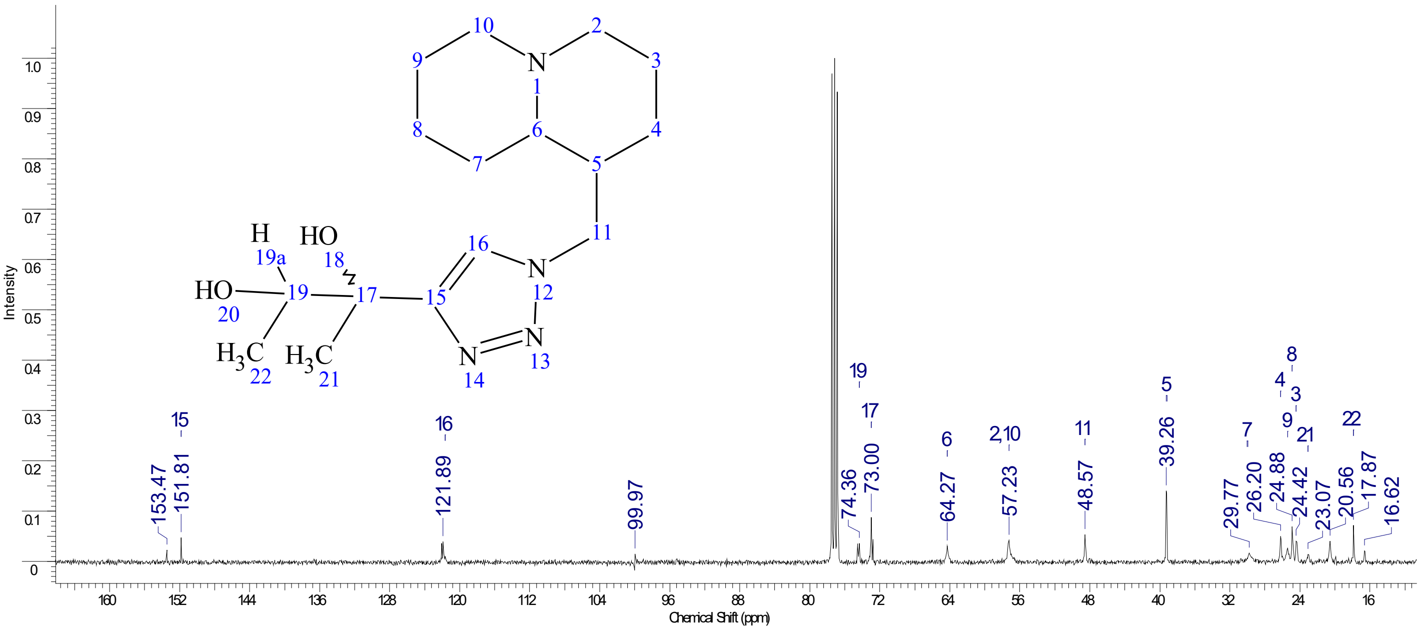

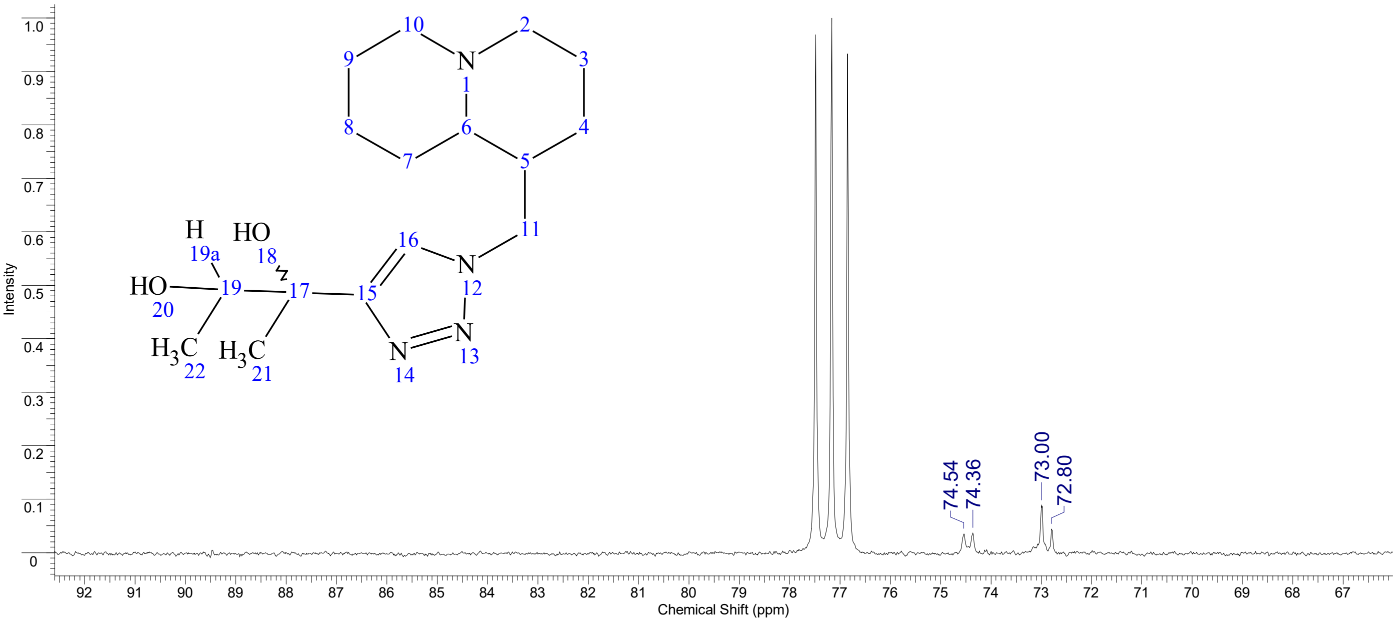

Supplementary Figure S5.3. The mass spectrum of (2*R*,*S*)-2-(1-(((1*S*,9a*R*)-octahydro-1*H*-quinolizine-1-yl)methyl)-1*H*-1,2,3-triazole-4-yl)butane-2,3-diol (6a,b).

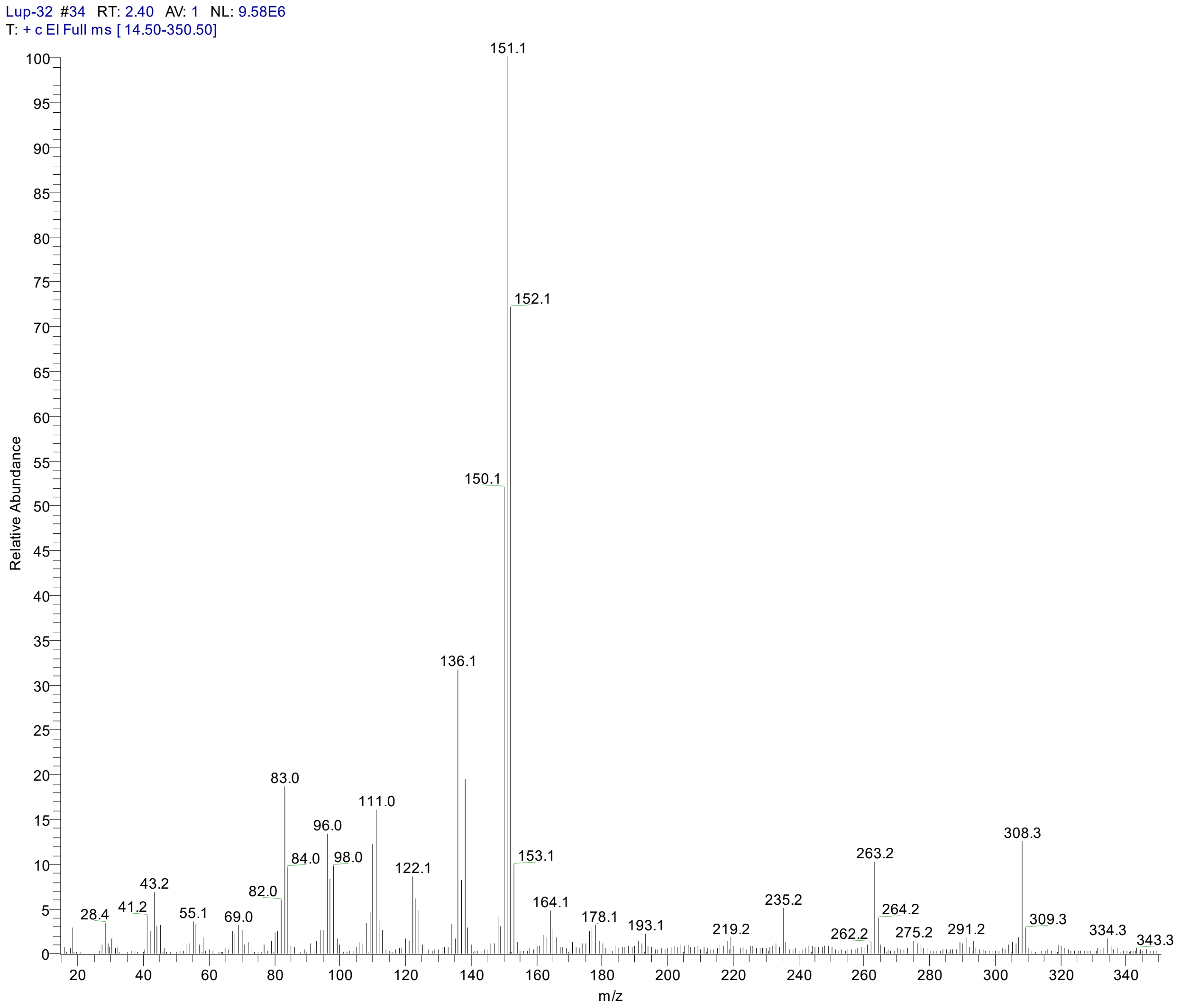

Supplementary Figure S6.1. ^{1}H NMR spectrum of 3-ethoxy-4-((1-(((1*S*,9a*R*)-octahydro-1*H*-quinolizine-1-yl)methyl)-1*H*-1,2,3-triazole-4-yl)methoxy)benzaldehyde (7).

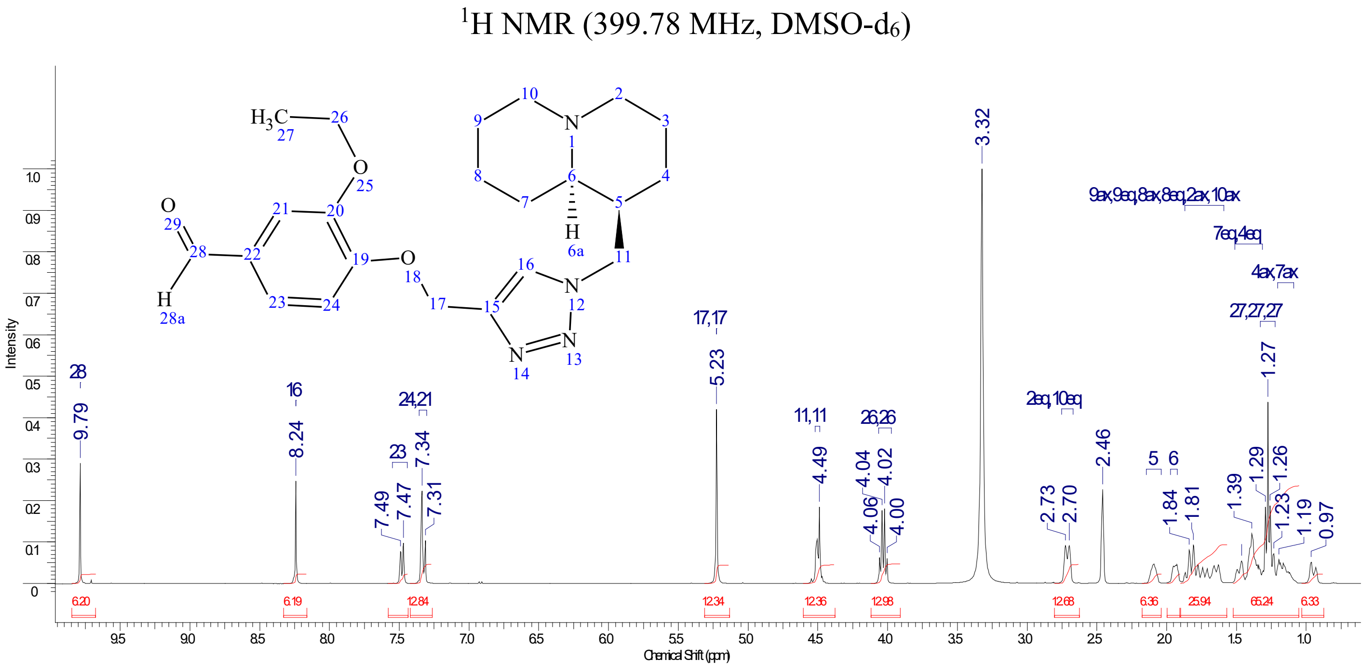

Supplementary Figure S6.2. ^{13}C NMR spectrum of 3-ethoxy-4-((1-(((1*S*,9a*R*)-octahydro-1*H*-quinolizine-1-yl)methyl)-1*H*-1,2,3-triazole-4-yl)methoxy)benzaldehyde (7).

^{13}C NMR (100.53 MHz, DMSO-d$_6$)

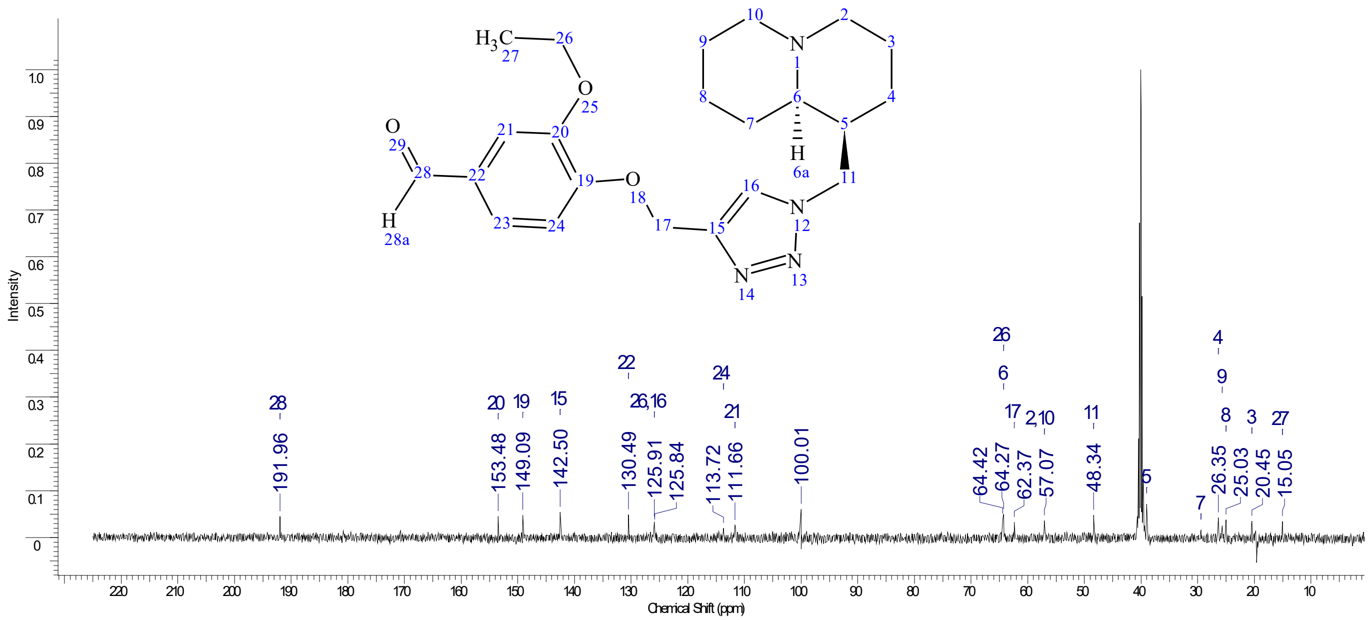

Supplementary Figure S6.3. The mass spectrum of 3-ethoxy-4-((1-(((1*S*,9a*R*)-octahydro-1*H*-quinolizine-1-yl)methyl)-1*H*-1,2,3-triazole-4-yl)methoxy)benzaldehyde (7).

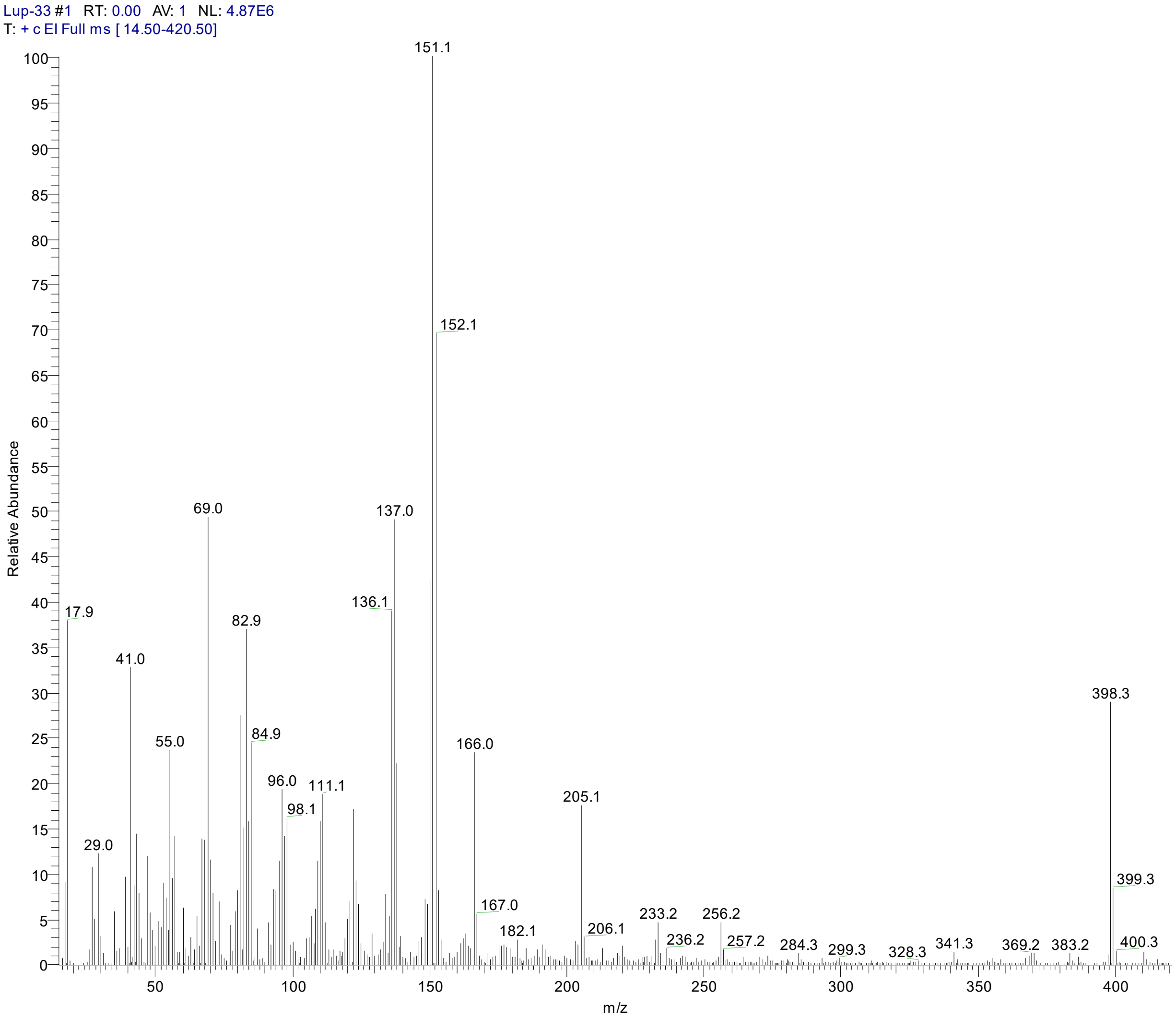

Supplementary Table S1. Chemical structures of lupinine-based esters of different carboxylic acids under investigation (Vitas-M codes are indicated)

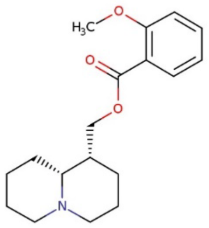 **18** (STOCK1N-00815)	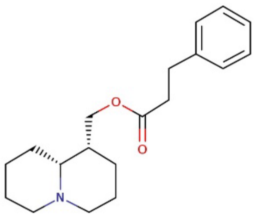 **19** (STOCK1N-20764)	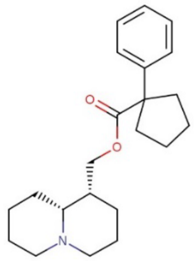 **20** (STOCK1N-27463)
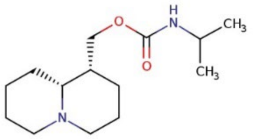 **21** (STOCK1N-08226)	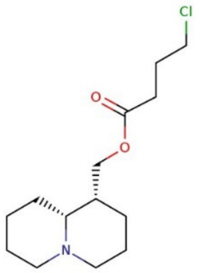 **22** (STOCK1N-20827)	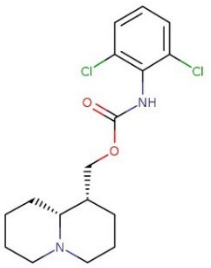 **23** (STOCK1N-27506)
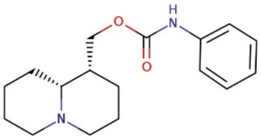 **24** (STOCK1N-09194)	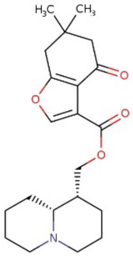 **25** (STOCK1N-20963)	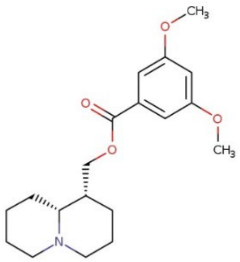 **26** (STOCK1N-27613)
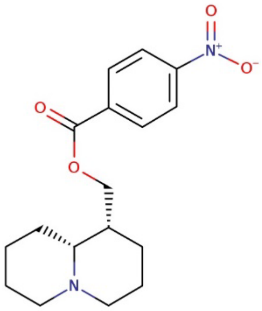 **27** (STOCK1N-11775)	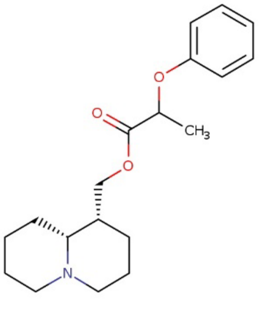 **28** (STOCK1N-21190)	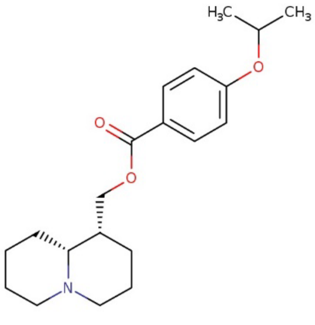 **29** (STOCK1N-27767)
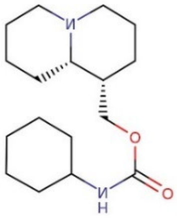 **30** (STOCK1N-11783)	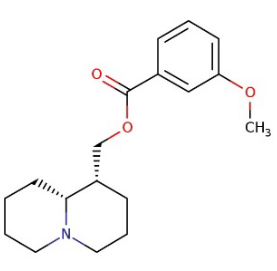	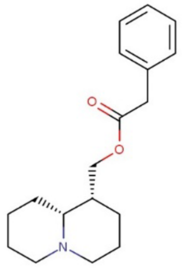

	**31** (STOCK1N-21225)	**32** (STOCK1N-28017)
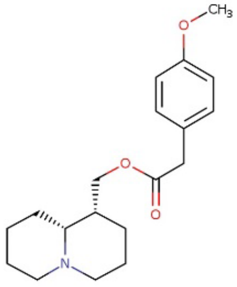 **33** (STOCK1N-16368)	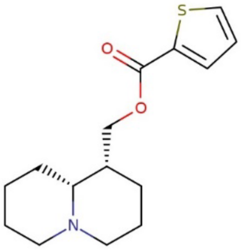 **34** (STOCK1N-22221)	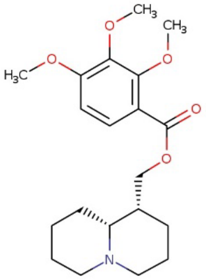 **35** (STOCK1N-28128)
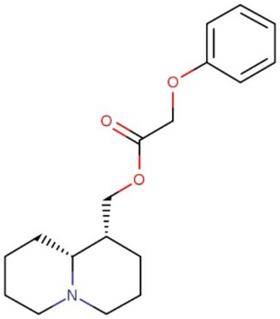 **36** (STOCK1N-17850)	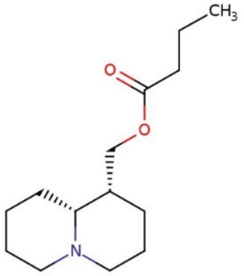 **37** (STOCK1N-22377)	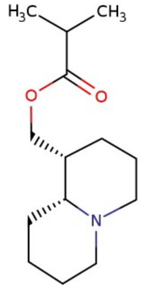 **38** (STOCK1N-28515)
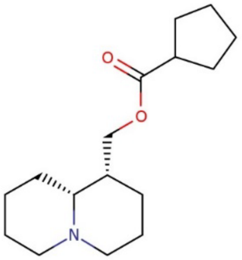 **39** (STOCK1N-18175)	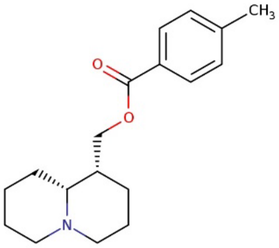 **40** (STOCK1N-23538)	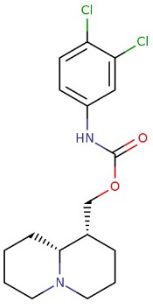 **41** (STOCK1N-29877)
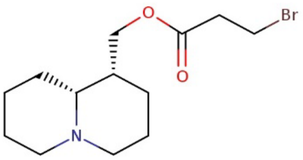 **42** (STOCK1N-18223)	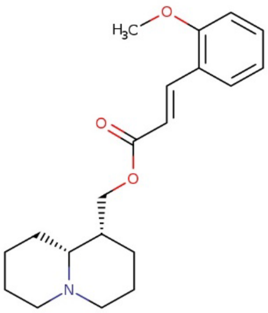 **43** (STOCK1N-25368)	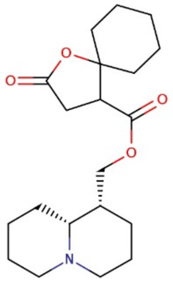 **44** (STOCK1N-30716)

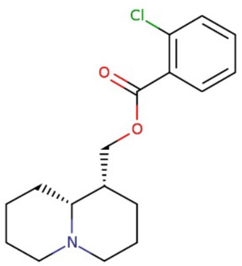 **45** (STOCK1N-18525)	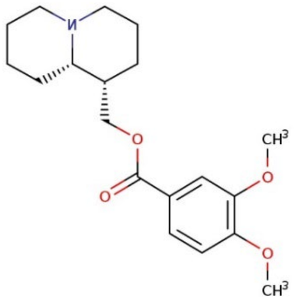 **46** (STOCK1N-25694)	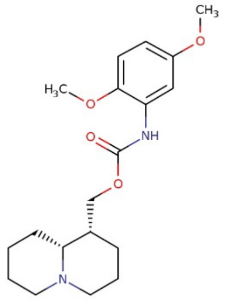 **47** (STOCK1N-32617)
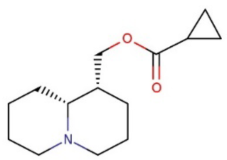 **48** (STOCK1N-18721)	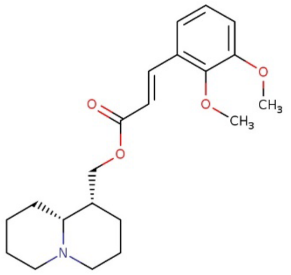 **49** (STOCK1N-25897)	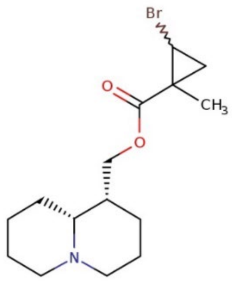 **50** (STOCK1N-41979)
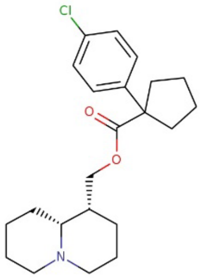 **51** (STOCK1N-18802)	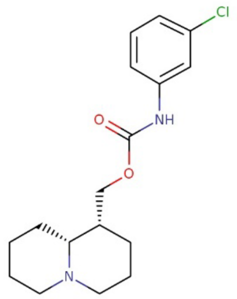 **52** (STOCK1N-25964)	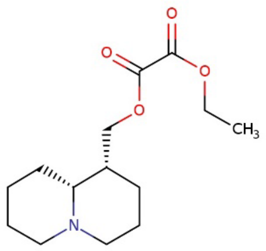 **53** (STOCK1N-42122)
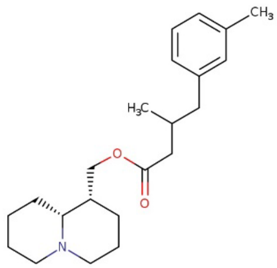 **54** (STOCK1N-19758)	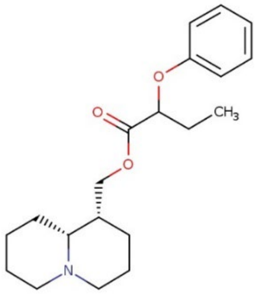 **55** (STOCK1N-27190)	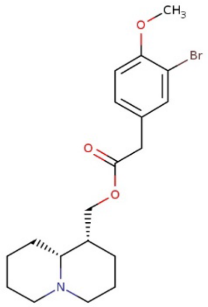 **56** (STOCK1N-42244)
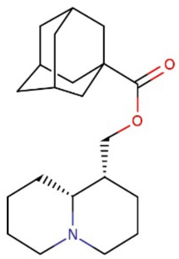 **57** (STOCK1N-20315)	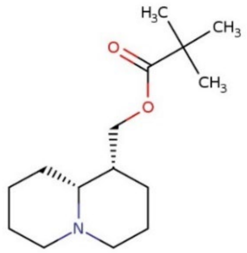 **58** (STOCK1N-27196)	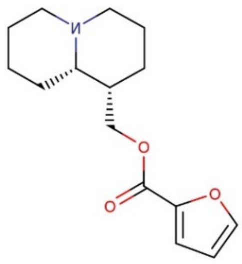 **59** (STOCK1N-42364)

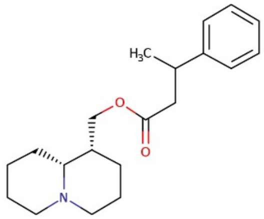<br>**60** (STOCK1N-20499)	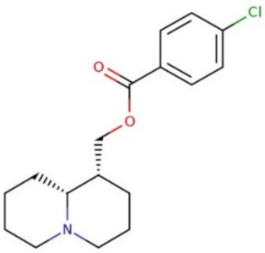<br>**61** (STOCK1N-27242)	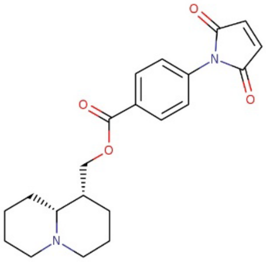<br>**62** (STOCK1N-43209)
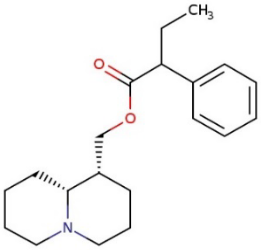<br>**63** (STOCK1N-20557)	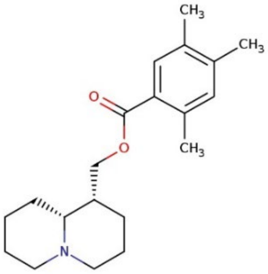<br>**64** (STOCK1N-27249)	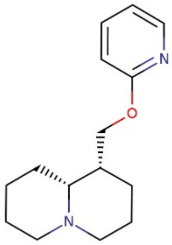<br>**65** (STOCK1N-43872)
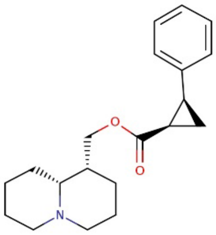<br>**66** (STOCK1N-20682)	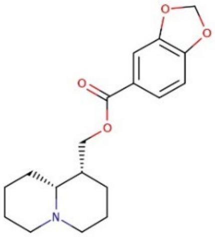<br>**67** (STOCK1N-27417)	

Supplementary Figure S7. 2D diagram of ligand-receptor interactions obtained on docking of compound 15 in AChE. Blue dashed lines – hydrogen bonding interactions. Red dashed line – steric interaction.

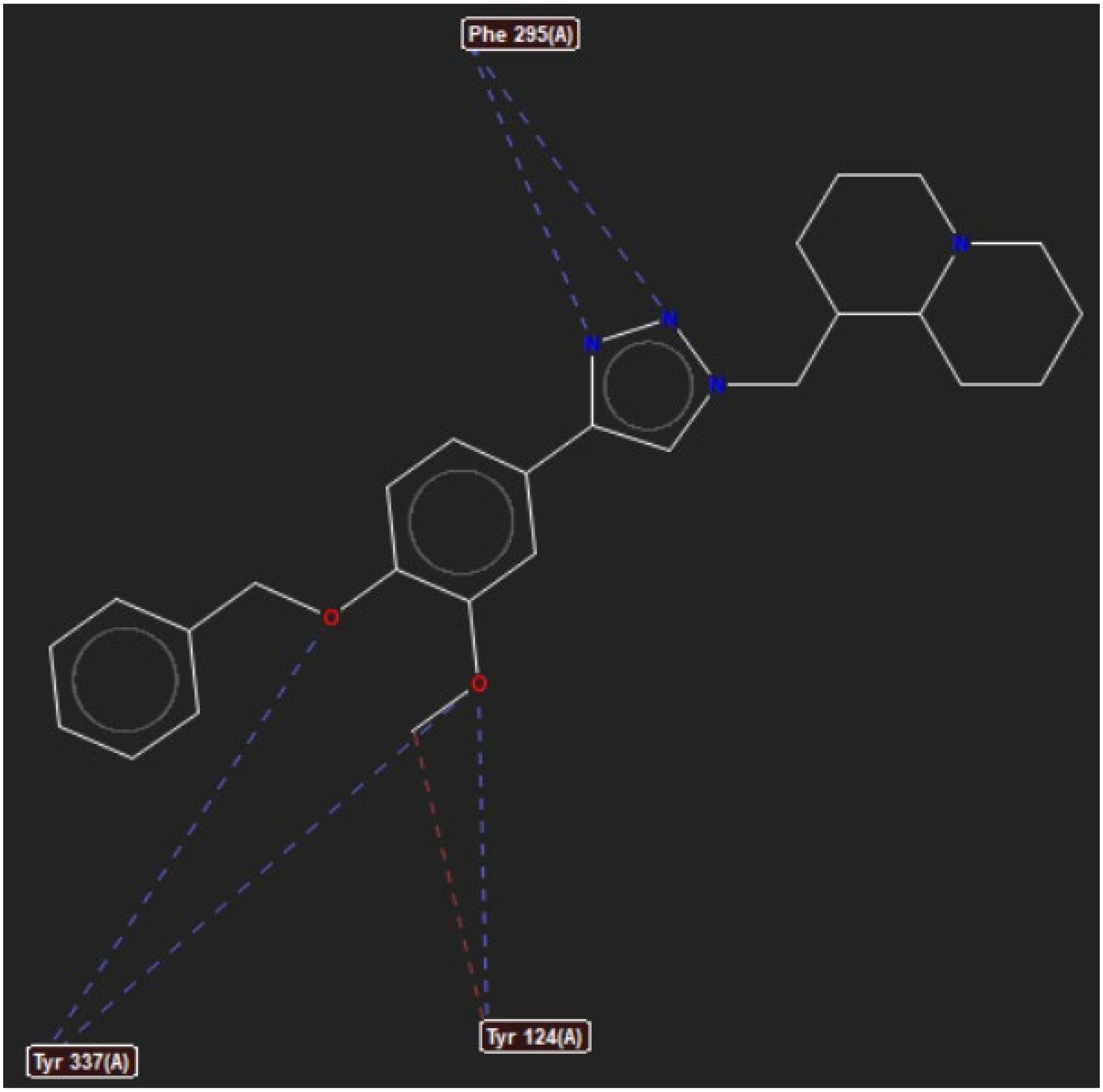

Supplementary Table S2. Chemical formulas, selected ADME parameters of the lupinine-based esters calculated with SwissADME web tool, and manually added indicator variable for the quaternary sp^3 carbon atom (Q).

Compound	Formula	MW	N_{rot}	MR	Silicos-IT $LogS_w$	Q
**18**	$C_{18}H_{25}NO_3$	303.4	5	90.06	-3.98	0
**19**	$C_{19}H_{27}NO_2$	301.42	6	92.96	-4.66	0
**20**	$C_{22}H_{31}NO_2$	341.49	5	105.15	-5.21	1
**21**	$C_{14}H_{26}N_2O_2$	254.37	5	76.47	-2.23	0
**22**	$C_{14}H_{24}ClNO_2$	273.8	6	78.08	-3.18	0
**23**	$C_{17}H_{22}Cl_2N_2O_2$	357.27	5	98.09	-5.1	0
**24**	$C_{17}H_{24}N_2O_2$	288.38	5	88.07	-3.91	0
**25**	$C_{21}H_{29}NO_4$	359.46	4	103.04	-4.79	0
**26**	$C_{19}H_{27}NO_4$	333.42	6	96.55	-4.09	0
**27**	$C_{17}H_{22}N_2O_4$	318.37	5	92.39	-3.22	0
**28**	$C_{19}H_{27}NO_3$	317.42	6	94.49	-4.02	0
**29**	$C_{20}H_{29}NO_3$	331.45	6	99.67	-4.4	0
**30**	$C_{17}H_{30}N_2O_2$	294.43	5	88.78	-2.83	0
**31**	$C_{18}H_{25}NO_3$	303.4	5	90.06	-3.98	0
**32**	$C_{18}H_{25}NO_2$	287.4	5	88.15	-4.26	0
**33**	$C_{19}H_{27}NO_3$	317.42	6	94.64	-4.38	0
**34**	$C_{15}H_{21}NO_2S$	279.4	4	81.44	-3.13	0
**35**	$C_{20}H_{29}NO_5$	363.45	7	103.04	-4.2	0
**36**	$C_{18}H_{25}NO_3$	303.4	6	89.68	-4	0
**37**	$C_{14}H_{25}NO_2$	239.35	5	73.28	-2.55	0
**38**	$C_{14}H_{25}NO_2$	239.35	4	73.28	-2.18	0
**39**	$C_{16}H_{27}NO_2$	265.39	4	80.78	-2.51	0
**40**	$C_{18}H_{25}NO_2$	287.4	4	88.53	-4.25	0
**41**	$C_{17}H_{22}Cl_2N_2O_2$	357.27	5	98.09	-5.1	0
**42**	$C_{13}H_{22}BrNO_2$	304.22	5	76.34	-3	0
**43**	$C_{20}H_{27}NO_3$	329.43	6	99.77	-4.05	0
**44**	$C_{20}H_{31}NO_4$	349.46	4	99.22	-3.19	0
**45**	$C_{17}H_{22}ClNO_2$	307.82	4	88.58	-4.46	0
**46**	$C_{19}H_{27}NO_4$	333.42	6	96.55	-4.09	0
**47**	$C_{19}H_{28}N_2O_4$	348.44	7	101.06	-4.13	0
**48**	$C_{14}H_{23}NO_2$	237.34	4	71.17	-1.95	0
**49**	$C_{21}H_{29}NO_4$	359.46	7	106.26	-4.16	0
**50**	$C_{15}H_{24}BrNO_2$	330.26	4	83.58	-3.15	1
**51**	$C_{22}H_{30}ClNO_2$	375.93	5	110.16	-5.8	1
**52**	$C_{17}H_{23}ClN_2O_2$	322.83	5	93.08	-4.51	0
**53**	$C_{14}H_{23}NO_4$	269.34	6	74.56	-1.83	0
**54**	$C_{22}H_{33}NO_2$	343.5	7	107.54	-5.46	0

**55**	$C_{20}H_{29}NO_3$	331.45	7	99.29	-4.42	0
**56**	$C_{19}H_{26}BrNO_3$	396.32	6	102.34	-5.17	0
**57**	$C_{21}H_{33}NO_2$	331.49	4	100.33	-3.38	1
**58**	$C_{15}H_{27}NO_2$	253.38	4	77.83	-2.56	1
**59**	$C_{15}H_{21}NO_3$	263.33	4	75.83	-3.08	0
**60**	$C_{20}H_{29}NO_2$	315.45	6	97.77	-4.68	0
**61**	$C_{17}H_{22}ClNO_2$	307.82	4	88.58	-4.46	0
**62**	$C_{21}H_{24}N_2O_4$	368.43	5	108.14	-3.57	0
**63**	$C_{20}H_{29}NO_2$	315.45	6	97.77	-4.68	0
**64**	$C_{20}H_{29}NO_2$	315.45	4	98.47	-5.01	0
**65**	$C_{15}H_{22}N_2O$	246.35	3	76.57	-3.55	0
**66**	$C_{20}H_{27}NO_2$	313.43	5	95.65	-4.21	0
**67**	$C_{18}H_{23}NO_4$	317.38	4	89.63	-3.6	0

Abbreviations: molecular weight (MW), number of rotatable bonds (N_{rot}), molar refraction (MR), and water solubility characteristic (SILICOS-IT Log S_w).

Supplementary Table S3. Experimentally determined, calculated, and LOO-predicted classes for AChE inhibitory activity of the lupinine-based esters of different carboxylic acids.

**Compound**	**Observed**	**LDA**	
		**Calculated**	**LOO-Predicted**
**18**	NA	NA	NA
**19**	NA	NA	NA
**20**	NA	NA	NA
**21**	NA	NA	NA
**22**	Active	NA	NA
**23**	NA	Active	Active
**24**	NA	NA	NA
**25**	Active	Active	Active
**26**	NA	NA	NA
**27**	NA	Active	Active
**28**	NA	NA	NA
**29**	NA	Active	Active
**30**	NA	NA	NA
**31**	NA	NA	NA
**32**	NA	NA	NA
**33**	NA	NA	NA

**34**	NA	NA	NA
**35**	NA	Active	Active
**36**	NA	NA	NA
**37**	NA	NA	NA
**38**	NA	NA	NA
**39**	NA	NA	NA
**40**	NA	NA	NA
**41**	Active	Active	NA
**42**	NA	NA	NA
**43**	Active	Active	Active
**44**	Active	Active	Active
**45**	NA	NA	Active
**46**	NA	NA	NA
**47**	NA	NA	Active
**48**	NA	NA	NA
**49**	Active	Active	Active
**50**	NA	NA	NA
**51**	NA	NA	NA
**52**	NA	NA	NA
**53**	NA	NA	NA
**54**	NA	NA	NA
**55**	NA	NA	NA
**56**	NA	Active	Active
**57**	NA	NA	NA
**58**	NA	NA	NA
**59**	NA	NA	NA
**60**	NA	NA	NA
**61**	NA	NA	Active
**62**	NA	Active	Active
**63**	NA	NA	NA
**64**	Active	Active	Active
**65**	NA	NA	NA
**66**	NA	Active	Active
**67**	NA	Active	Active
